# Eusiridae (Crustacea, Amphipoda) of the Clarion-Clipperton Zone in the abyssal east Pacific with descriptions of five new species

**DOI:** 10.3897/zookeys.1274.140610

**Published:** 2026-03-24

**Authors:** Anne-Nina Lörz, Laura Engel, Halina Jereczek, Nikodem Ćwierz, Anna M. Jażdżewska

**Affiliations:** 1 Marine Research at Senckenberg am Meer, Südstrand 40, 26832 Wilhelmshaven, Germany Center for Earth System Research and Sustainability (CEN), Institute of Marine Ecosystem and Fishery Science (IMF), University of Hamburg Hamburg Germany; 2 Center for Earth System Research and Sustainability (CEN), Institute of Marine Ecosystem and Fishery Science (IMF), University of Hamburg, Große Elbstraße 133, 22767 Hamburg, Germany Marine Research at Senckenberg am Meer Wilhelmshaven Germany; 3 Department of Invertebrate Zoology and Hydrobiology, Faculty of Biology and Environmental Protection, University of Lodz, Banacha 12/16, 90-237 Lodz, Poland Department of Invertebrate Zoology and Hydrobiology, Faculty of Biology and Environmental Protection, University of Lodz Lodz Poland

**Keywords:** *
Cleonardo
*, deep sea, *
Dorotea
*, integrative taxonomy, *
Rhachotropis
*

## Abstract

Eusiridae is the most abundant and species rich family of Amphipoda collected in the Clarion-Clipperton Zone (CCZ), in the abyssal Eastern Pacific. Here five species new to science are described: *Cleonardo daniela***sp. nov**., *Cleonardo compassionate***sp. nov**., *Dorotea elizae***sp. nov**., *Rhachotropis clarionclippertoni***sp. nov**., and *Rhachotropis laure***sp. nov**. In addition to detailed illustrations, molecular barcodes and confocal laser scanning microscope (CLSM) images are provided of the new species described. All amphipod taxa from the Clarion-Clipperton Zone were collected at abyssal depths, greater than 4000 metres.

## Introduction

Amphipoda of the family Eusiridae are fast-moving predators with a worldwide distribution (Lörz et al. 2023). In the bathyal and abyssal zones they constitute a diverse and often abundant component of suprabenthic assemblages (Brix et al. 2018; Jażdżewska and Mamos 2019). The family consists of 13 genera and 131 species (Horton et al. 2024). The study of the Amphipoda collected in the abyssal zone in the Clarion-Clipperton Zone (CCZ) revealed the family Eusiridae to be one of two dominant taxa in terms of abundance and species richness (Jażdżewska et al. 2025). Morphological study of the cited collection resulted in the identification of several species new to science; two *Cleonardo* Stebbing, 1888, two *Rhachotropis* S.I. Smith, 1883 and one *Dorotea* Corbari, Frutos & Sorbe, 2019. The eusirid species new to science were sampled in the area of manganese nodule fields of the Clarion-Clipperton Zone below 4000 m depth.

## Material and methods

The material for study was sampled in the central east Pacific, specifically in the easternmost sector of the Clarion-Clipperton Zone (CCZ). The material was collected with an epibenthic sledge (EBS) during four scientific deep-sea cruises: the ABYSSLINE-2 (ABYSSal baseLINE project) in 2015, MANGAN 2016, MANGAN 2018, and MANGAN 2023 (Table 1). One individual collected during KuramBio (Kurile-Kamchatka Biodiversity Study) expedition that appeared molecularly as belonging to one of the described species was also included (Table 1). For details of gear deployment and sample processing see Jażdżewska and Horton (2026) and Brandt et al. (2015).

**Table 1.** Station data. Exploration contract area (ECA): UKSR-1 – UK Seabed Resources Ltd, United Kingdom, OMS – Ocean Mineral Singapore Pte. Ltd., BGR – Bundesanstalt für Geowissenschaften und Rohstoffe; APEI 6 – Area of Particular Environmental Interest 6.

**Expedition**	**Vessel**	**ECA**	**Site code**	**Latitude, Longitude**	**Depth [m]**	**Collection date**
ABYSSLINE-2	R/V Thompson	UKSR-1	AB2-EB01	12°22.02'N, 116°33'W	4209	18-Feb-2015
ABYSSLINE-2	R/V Thompson	OMS	AB2-EB06	12°15.06'N, 117°19.2'W	4137	01-Mar-2015
ABYSSLINE-2	R/V Thompson	UKSR-1	AB2-EB09	12°21.6'N, 116°42'W	4170	10-Mar-2015
ABYSSLINE-2	R/V Thompson	OMS	AB2-EB11	12°2.28'N, 117°14.22'W	4097	14-Mar-2015
ABYSSLINE-2	R/V Thompson	APEI 6	AB2-EB13	19°27.9'N, 120°1.5'W	4026	20-Mar-2015
MANGAN 2016	R/V Kilo Moana	BGR	Ma 16-18	11°51.372'N–11°51.662'N, 117°01.535'W–117°00.482'W	4132–4123	28-Apr-2016
MANGAN 2016	R/V Kilo Moana	BGR	Ma 16-28	11°49.654'N–11°49.902'N, 117°00.299'W–116°59.174'W	4143–4133	01-May-2016
MANGAN 2016	R/V Kilo Moana	BGR	Ma 16-91	11°49.792'N–11°49.842'N, 117°30.458'W–117°29.208'W	4344–4344	09-May-2016
MANGAN 2016	R/V Kilo Moana	BGR	Ma 16-95	11°47.862'N–11°47.152'N, 117°30.639'W–117°29.490'W	4356–4359	09-May-2016
MANGAN 2018	R/V Sonne	BGR	SO 262-150	11°50.009'N–11°49.978'N, 116°14.780'W–116°13.316'W	4074–4095	07-May-2018
MANGAN 2018	R/V Sonne	BGR	SO 262-155	11°47.436'N–11°47.677'N, 117°32.213'W–117°30.910'W	4352–4351	09-May-2018
MANGAN 2018	R/V Sonne	BGR	SO 262-156	11°49.381'N–11°49.752'N, 117°32.663'W–117°30.760'W	4340–4340	09-May-2018
MANGAN 2018	R/V Sonne	BGR	SO 262-59	11°49.720'N–11°50.055'N, 117°01.080'W–116°59.530'W	4097–4128	22-Apr-2018
MANGAN 2018	R/V Sonne	BGR	SO 262-67	11°51.190'N–11°51.621'N, 117°02.830'W–117°00.804'W	4131–4131	24-Apr-2018
MANGAN 2023	R/V Kilo Moana	BGR	KM23-49	11°45.4904'N–11°46.3035'N, 116°50.4565'W–116°49.3268'W	4150–4173	30-Apr-2023
MANGAN 2023	R/V Kilo Moana	BGR	KM23-50	11°17.7919'N–11°18.5445'N, 116°18.8626'W–116°17.6747'W	4182–4185	01-May-2023
MANGAN 2023	R/V Kilo Moana	BGR	KM23-69	11°36.252'N–11°37.1050'N, 118°02.981'W–118°01.2511'W	4368–4356	04-May-2023
MANGAN 2023	R/V Kilo Moana	BGR	KM23-74	11°47.6444'N–11°48.0414'N, 117°30.9650'W–117°29.5413'W	4360–4364	06-May-2023
MANGAN 2023	R/V Kilo Moana	BGR	KM23-79	11°51.3560'N–11°51.7516'N, 117°01.2662'W–116°59.8924'W	4126–4128	07-May-2023
MANGAN 2023	R/V Kilo Moana	BGR	KM23-93	12°06.612'N–12°07.3536'N, 119°01.072'W–118°59.8503'W	4381–4430	10-May-2023
KuramBio	R/V Sonne	N/A	SO 223-7-9	43°01.78'N–43°01.49'N, 152°58.61'E–152°58.36'E	5222–5223	17-Aug-2012

Individuals were initially examined using either a Leica M125 or a Nikon SMZ800 stereomicroscope. Hand drawings of the habitus were prepared using a Nikon SMZ1500 stereomicroscope equipped with a camera lucida. When possible, the habitus is presented as photographs obtained with a confocal laser scanning microscope (CLSM). The holotypes were stained in Congo red and acid fuchsin, temporarily mounted onto slides with glycerine and examined with a Leica TCS SPV equipped with a Leica DM5000 B upright microscope and three visible-light lasers (DPSS 10 mW 561 nm; HeNe 10 mW 633 nm; Ar 100 mW 458, 476, 488 and 514 nm), combined with the software LAS AF 2.2.1 (Leica Application Suite, Advanced Fluorescence). A series of photographic stacks were obtained, collecting overlapping optical sections throughout the whole preparation (Michels and Büntzow 2010; Kamanli et al. 2017). Type material was dissected and mounted on permanent slides using polyvinyl-lactophenol containing lignin pink. All slides were examined using either a Nikon Eclipse Ci or Zeiss compound microscope equipped with a camera lucida. Pencil drawings from the microscope were used as the basis for line drawings. The drawings were inked with CorelDraw or Adobe Illustrator CS6 following the recommendations of Coleman (2003, 2009).

The following abbreviations are used: **A1, 2** = antenna 1, 2; **AF** = accessory flagellum; **H** = head; **UL** = upper lip; **LL** = lower lip; **Md** = mandible; **Mx1, 2** = maxilla 1, 2; **Mxp** = maxilliped; **c1–4** = coxa 1–4; **G1, 2** = gnathopod 1, 2; **P3–7** = pereopod 3–7; **pl1–3** = pleopod 1–3; **U1–3** = uropod 1–3; **T** = telson; **l** = left; **r** = right.

The registered type material is deposited in the Senckenberg Museum (Frankfurt, Germany) (**SMF**).

All the remaining material is kept in Deutsches Zentrum für Marine Biodiversitätsforschung (**DZMB**) in Wilhelmshaven, Germany. All individuals were subjected to cytochrome *c* oxidase subunit I gene (COI) barcoding prior to identification of the species. The molecular procedures are described in Jażdżewska et al. (2025). All sequences were deposited in Barcode of Life Data System (BOLD) and in GenBank (Table 2). The Relevant voucher information, taxonomic classifications, and sequences are deposited in the data set “DS-AMPHICCZ” in the Barcode of Life Data System (BOLD) (https://doi.org/10.5883/DS-AMPHICCZ) (www.boldsystems.org) (Ratnasingham and Hebert 2007).

**Table 2.** Summary of the material used in the present study. BOLD – Barcode of Life Data Systems, BIN – Barcode Index Number, DZMB – Deutsches Zentrum für Marine Biodiversitätsforschung, Wilhelmshaven, Germany, SMF – Senckenberg Museum Frankfurt, Germany, UL – University of Lodz, Poland.

**Sample ID**	**Type status**	**Sex**	**Size [mm]**	**Institution depository**	**Museum code**	**BOLD process ID**	**BIN**	**GenBank COI acc. number**	**Site code**
*** Cleonardo compassionate* sp. nov**.									
DSB_3776	holotype	mature female	10	SMF	SMF 62814	CCZ2941-19	BOLD:ADF7841	PQ734425	AB2-EB06
DSB_3640	paratype	mature male	10	SMF	SMF 62815	CCZ2843-19	BOLD:ADF7841	PQ734442	SO 262-150
DSB_3555	non type collection	juvenile	3.5	DZMB		CCZ2758-19	BOLD:ADF7841	PQ734478	SO 262-155
DSB_7958	non type collection	juvenile	3	DZMB		MNGN149-24	BOLD:ADF7841	PQ734528	KM23-50
7-9S_Eusi6_2012_2	non type collection	mature male	8	UL		AJAKK681-17	BOLD:ADF7841	MN346344	SO 223-7-9
*** Cleonardo daniela* sp. nov**.									
DSB_3741	holotype	mature male	6.5	SMF	SMF 62810	CCZ2906-19	BOLD:AEB2179	PQ734433	AB2-EB01
DSB_3800	paratype	immature male	7	SMF	SMF 62811	CCZ2965-19	BOLD:AEB2179	PQ734235	AB2-EB09
DSB_7955	paratype	mature male	12	SMF	SMF 62812	MNGN146-24	BOLD:AEB2179	PQ734289	KM23-50
DSB_7957	paratype	juvenile male	5.5	SMF	SMF 62813	MNGN148-24	BOLD:AEB2179	PQ734766	KM23-50
DSB_3563	non type collection	undetermined	undetermined	DZMB		CCZ2766-19	BOLD:AEB2179	PQ734632	SO 262-59
DSB_3564	non type collection	undetermined	undetermined	DZMB		CCZ2767-19	BOLD:AEB2179	PQ734509	SO 262-59
DSB_3565	non type collection	undetermined	undetermined	DZMB		CCZ2768-19	BOLD:AEB2179	PQ734246	SO 262-59
DSB_7960	non type collection	juvenile	3	DZMB		MNGN151-24	BOLD:AEB2179	PQ734545	KM23-50
DSB_7961	non type collection	juvenile	3	DZMB		MNGN152-24	BOLD:AEB2179	PQ734271	KM23-50
DSB_7962	non type collection	juvenile	3	DZMB		MNGN153-24	BOLD:AEB2179	PQ734428	KM23-50
DSB_7963	non type collection	juvenile	3	DZMB		MNGN154-24	BOLD:AEB2179	PQ734687	KM23-50
*** Dorotea elizae* sp. nov**.									
DSB_3775	holotype	male	16	SMF	SMF 62816	CCZ2940-19	BOLD:AEB4129	PQ734718	AB2-EB06
*** Rhachotropis clarionclippertoni* sp. nov**.									
DSB_3660	holotype	female	8	SMF	SMF 62817	CCZ2668-19	BOLD:AEB0147	PQ734529	Ma 16-91
DSB_3658	paratype	female	8.5	SMF	SMF 62818	CCZ2666-19	BOLD:AEB0147	PQ734352	Ma 16-91
DSB_3656		undetermined	undetermined	DZMB		CCZ2664-19	BOLD:AEB0147	PQ734730	Ma 16-28
DSB_3659		undetermined	undetermined	DZMB		CCZ2667-19	BOLD:AEB0147	PQ734541	Ma 16-91
DSB_3661		undetermined	undetermined	DZMB		CCZ2669-19	BOLD:AEB0147	PQ734532	Ma 16-91
DSB_3662		female	undetermined	DZMB		CCZ2670-19	BOLD:AEB0147	PQ734453	Ma 16-91
DSB_3663		female	undetermined	DZMB		CCZ2671-19	BOLD:AEB0147	PQ734732	Ma 16-91
DSB_3664		juvenile	undetermined	DZMB		CCZ2672-19	BOLD:AEB0147	PQ734743	Ma 16-91
DSB_3665		undetermined	undetermined	DZMB		CCZ2673-19	BOLD:AEB0147	PQ734323	Ma 16-91
DSB_3667		undetermined	undetermined	DZMB		CCZ2675-19	BOLD:AEB0147	PQ734299	Ma 16-95
DSB_3669		undetermined	undetermined	DZMB		CCZ2677-19	BOLD:AEB0147	PQ734277	Ma 16-95
DSB_3672		juvenile	undetermined	DZMB		CCZ2680-19	BOLD:AEB0147	PQ734782	Ma 16-18
DSB_3558		female	undetermined	DZMB		CCZ2761-19	BOLD:AEB0147	PQ734715	SO 262-156
DSB_3559		female	undetermined	DZMB		CCZ2762-19	BOLD:AEB0147	PQ734550	SO 262-156
DSB_3562		female	undetermined	DZMB		CCZ2765-19	BOLD:AEB0147	PQ734542	SO 262-59
DSB_3567		female	undetermined	DZMB		CCZ2770-19	BOLD:AEB0147	PQ734336	SO 262-67
DSB_7868		undetermined	undetermined	DZMB		MNGN059-24	BOLD:AEB0147	PQ734416	KM23-74
DSB_7880		undetermined	undetermined	DZMB		MNGN071-24	BOLD:AEB0147	PQ734287	KM23-74
DSB_7881		undetermined	undetermined	DZMB		MNGN072-24	BOLD:AEB0147	PQ734397	KM23-74
DSB_7891		undetermined	undetermined	DZMB		MNGN082-24	BOLD:AEB0147	PQ734783	KM23-79
DSB_8327		undetermined	undetermined	DZMB		MNGN508-24	BOLD:AEB0147	PQ734309	KM23-93
DSB_8337		undetermined	undetermined	DZMB		MNGN518-24	BOLD:AEB0147	PQ734574	KM23-93
*** Rhachotropis laure* sp. nov**.									
DSB_3655	holotype	female	10.5	DZMB	SMF 62819	CCZ2663-19	BOLD:AEB2577	PQ734767	Ma 16-28
DSB_3552	paratype	female	10	DZMB	SMF 62820	CCZ2755-19	BOLD:AEB2577	PQ734754	SO 262-155
DSB_3553	non type collection	female	undetermined	DZMB		CCZ2756-19	BOLD:AEB2577	PQ734430	SO 262-155
DSB_3557	non type collection	undetermined	undetermined	DZMB		CCZ2760-19	BOLD:AEB2577	PQ734414	SO 262-155
DSB_3568	non type collection	female	undetermined	DZMB		CCZ2771-19	BOLD:AEB2577	PQ734660	SO 262-67
DSB_3826	non type collection	female	undetermined	DZMB		CCZ2991-19	BOLD:AEB2577	PQ734661	AB2-EB13
DSB_7946	non type collection	undetermined	undetermined	DZMB		MNGN137-24	BOLD:AEB2577	PQ734477	KM23-50
DSB_7948	non type collection	undetermined	undetermined	DZMB		MNGN139-24	BOLD:AEB2577	PQ734375	KM23-50
DSB_8397	non type collection	undetermined	undetermined	DZMB		MNGN578-24	BOLD:AEB2577	PQ734562	KM23-69
DSB_8398	non type collection	undetermined	undetermined	DZMB		MNGN579-24	BOLD:AEB2577	PQ734726	KM23-69
DSB_8399	non type collection	undetermined	undetermined	DZMB		MNGN580-24	BOLD:AEB2577	PQ734755	KM23-69
DSB_8352	non type collection	undetermined	undetermined	DZMB		MNGN533-24	BOLD:AEB2577	PQ734320	KM23-49

## Results

### Systematics

**Order AMPHIPODA Latreille, 1816**

**Suborder AMPHILOCHIDEA Boeck, 1871**

**Family Eusiridae Stebbing, 1888**

#### Genus Cleonardo

Taxon classificationAnimaliaAmphipodaEusiridae

Stebbing, 1888

89E71B3D-794E-50D5-BE39-F7F8E7FDBD40

##### Type species.

*Cleonardo longipes* Stebbing, 1888.

#### Cleonardo daniela sp. nov.

Taxon classificationAnimaliaAmphipodaEusiridae

4C6552D5-A016-5A66-92A3-BC876E09E712

https://zoobank.org/07CA4D7B-5287-48CB-B1B8-93C46C6A2CD5

Figs 1, 2, 3, 4

##### Type material.

***Holotype***: Pacific • mature male, 6.5 mm, SMF 62810, COI: PQ734433, body remnants and one slide with appendages; Clarion-Clipperton Zone; UKSR-1 exploration contract area, R/V *Thompson*, ABYSSLINE-2, EBS, AB2-EB01, 18/02/2015, 12°22.02'N, 116°33'W, 4209 m. ***Paratypes***: Pacific • immature male, 7 mm, SMF 62811, COI: PQ734235, Clarion-Clipperton Zone; UKSR-1 exploration contract area, R/V *Thompson*, ABYSSLINE-2, EBS, AB2-EB09, 10/03/2015, 12°21.6'N, 116°42'W, 4170 m • two individuals: mature male, 12 mm, SMF 62812, COI: PQ734289, juvenile male, 5.5 mm, SMF 62813, COI: PQ734766, Clarion-Clipperton Zone; BGR exploration contract area, R/V *Kilo Moana*, MANGAN 2023, EBS, KM23-50, 01/05/2023, 11°17.7919'N, 116°18.8626'W–11°18.5445'N, 116°17.6747'W, 4185–4182 m.

##### Additional material.

Pacific • Clarion-Clipperton Zone; BGR exploration contract area, several stations (Table 2).

##### Type locality.

Abyssal Pacific Ocean, Clarion-Clipperton Zone, 12°22.02'N, 116°33'W, 4209 m.

##### Description.

**Figure 1.**
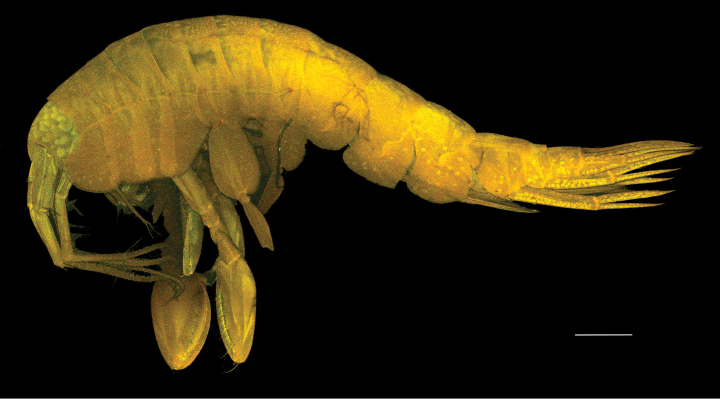
*
Cleonardo daniela* sp. nov., holotype, male, 6,5 mm, SMF 62810. Scale bar: 0.5 mm.

***Body*** (Fig. 1): dorsally smooth.

***Head*** (Fig. 1): rostrum acute and curved, reaching 1/3 of length of first article of peduncle of antenna 1; head lobes rounded and not covered by coxa 1.

***Eyes***: present, small, rounded, white in preserved specimen.

**Figure 2.**
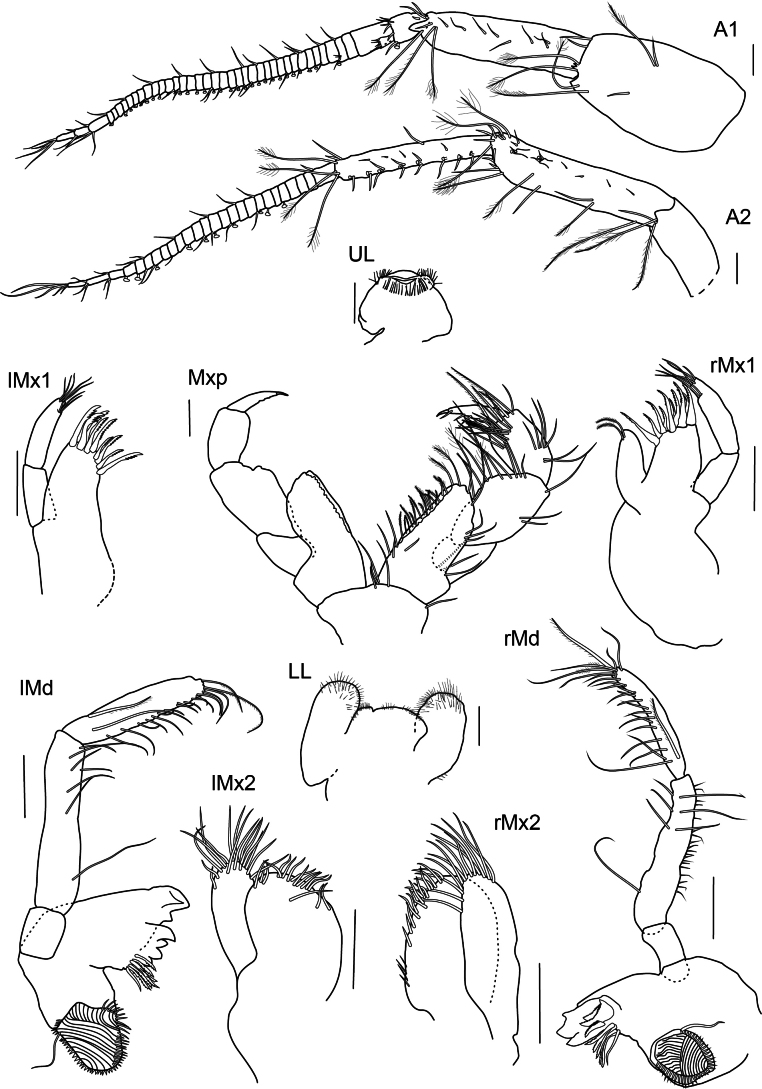
*
Cleonardo daniela* sp. nov., holotype, SMF 62810, male, 6.5 mm, Pacific, for clarity some spines and setae are omitted from the maxilliped. Scale bars: 0.1 mm.

***Antenna 1*** (Fig. 2): slightly shorter than antenna 2, weakly setose, reaching 1/2 the body; first article broad, extending to form two small teeth distally, same length as article 2; article 2 ending in acute extension distolaterally; few long plumose setae on article 1 and 2; article 3 shorter than first segment of flagellum with short setae; accessory flagellum uni-articulated, 1/2 the length of first segment of flagellum, with few thin setae on apex; flagellum 39-articulated, slightly longer than articles 1–3 combined, segments very narrow with first segment as long as the five subsequent ones, last three segments longer than previous ones, few setae on both sides and calceoli posteriorly.

***Antenna 2*** (Fig. 2): weakly setose, reaching 1/2 the body; peduncular article 3 1/2 the length of article 4; article 4 longer and broader than article 5; article 5 slender; articles 4 and 5 with short normal and long plumose setae; calceoli on posterior margin of article 5 and flagellum; article 4 only with two or three calceoli at lower half; flagellum shorter than articles 4 and 5 combined, with few setae and 26-articulated, first segment as long as nearly three of the subsequent ones and last three articles longer than the previous ones.

***Mandibles*** (Fig. 2): palp tri-articulated, article 3 shorter than article 2; articles 2 and 3 with long setae on inner margins and each with few plumose setae apical of article 3, second article of palp of right mandible additionally with many short setae on outer margin, article 3 additionally with one strong pectinated setae on lateral side and another one on apex; left lacinia mobilis 7-dentate, right lacinia 3-dentate and bifid; accessory spine row 6-dentate setae on the left and five on the right mandible, each with one additional normal long setae; molar triturative with dozens of denticles, and carrying a small plumose seta.

***Maxillae 1*** (Fig. 2): palp bi-articulated, exceeding outer lobe; outer lobe with nine or ten apical spine-teeth; inner lobe of left maxilla 1 missing; inner lobe of right maxilla not very broad, reaching length of outer lobe, apex with two plumose setae.

***Maxillae 2*** (Fig. 2): inner lobe 2× as broad as outer, reaching height of outer lobe; thick setae on inner lobe reaching from apex to nearly the end of front margin; apex of outer lobe with setae that are > 2× length of setae of inner lobe.

***Maxilliped*** (Fig. 2): inner lobes missing; outer lobe slightly serrated, with row of plumose setae on inner margins, reaching 1/2 the length of article 2 of palp, apex with four long plumose setae; palp four-articulated; article 2 broader and longer than other articles, inner margin with long setae; article 3 with rows of plumose setae; inner margin of dactylus with four setae-like spines.

***Upper lip*** (Fig. 2): entire, ventral margin broadly rounded, with fine setae.

***Lower lip*** (Fig. 2): broad, inner lobes weak, outer lobes larger; with many thin setae.

**Figure 3.**
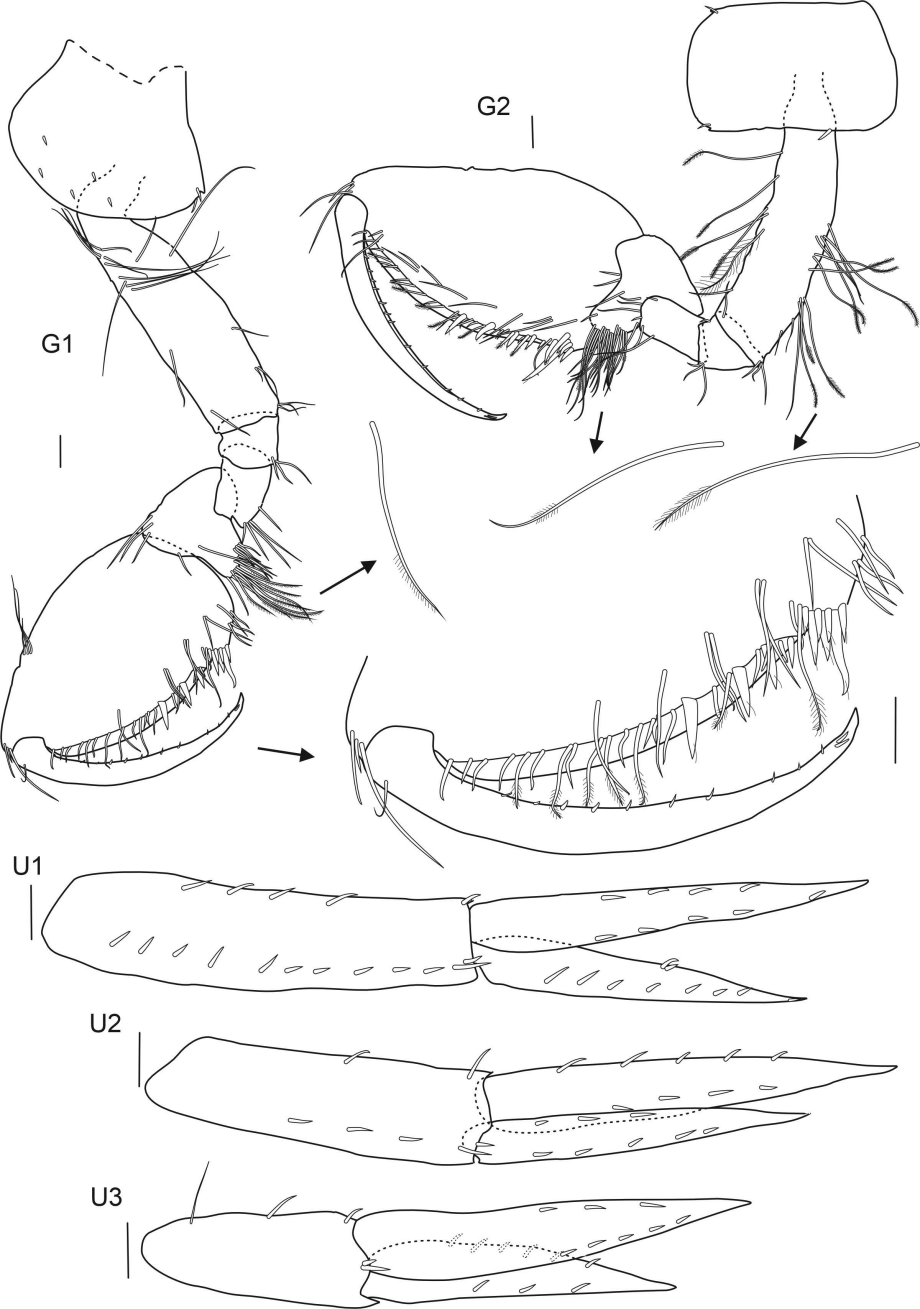
*
Cleonardo daniela* sp. nov., holotype, SMF 62810, male, 6.5 mm, Pacific. Scale bars: 0.1 mm

**Pereon: *Gnathopod 1*** (Fig. 3): slightly smaller than gnathopod 2 but of similar shape, both strongly subchelate; coxa broadly rounded anteriorly, strongly widening distally, axe shaped, not covering head lobes; posterior margin almost straight, with small notch armed with one spine, as deep as broad; basis elongated, with few long setae; ischium and merus short with few setae; carpus not broad, posterior edge with few setae, carpal lope slightly elongated with four rows of four or five pectinated setae; subovate propodus with tuft of setae on upper hind margin and apex, palm curved, with simple and plumose setae and four spines, palmar angle with seven spines with one very long; dactylus with ten small stout setae on inner margin and two longer ones on tip.

***Gnathopod 2*** (Fig. 3): coxa rectangular, nearly as long as coxa 1; anterior and posterior lower angles with little notch; posterior margin proximally with one strong spine, basis with more long plumose setae than gnathopod 1 and with one pappose seta; carpal lobe less elongated, with four rows of seven or eight plumose setae; propodus more elongated and upper hind margin nearly straight, palm similar to gnathopod 1 but with four spines, palmar angle with six spines with one 2× length of other spines; dactylus with 12 small stout setae on inner margin and two longer ones on tip.

**Figure 4.**
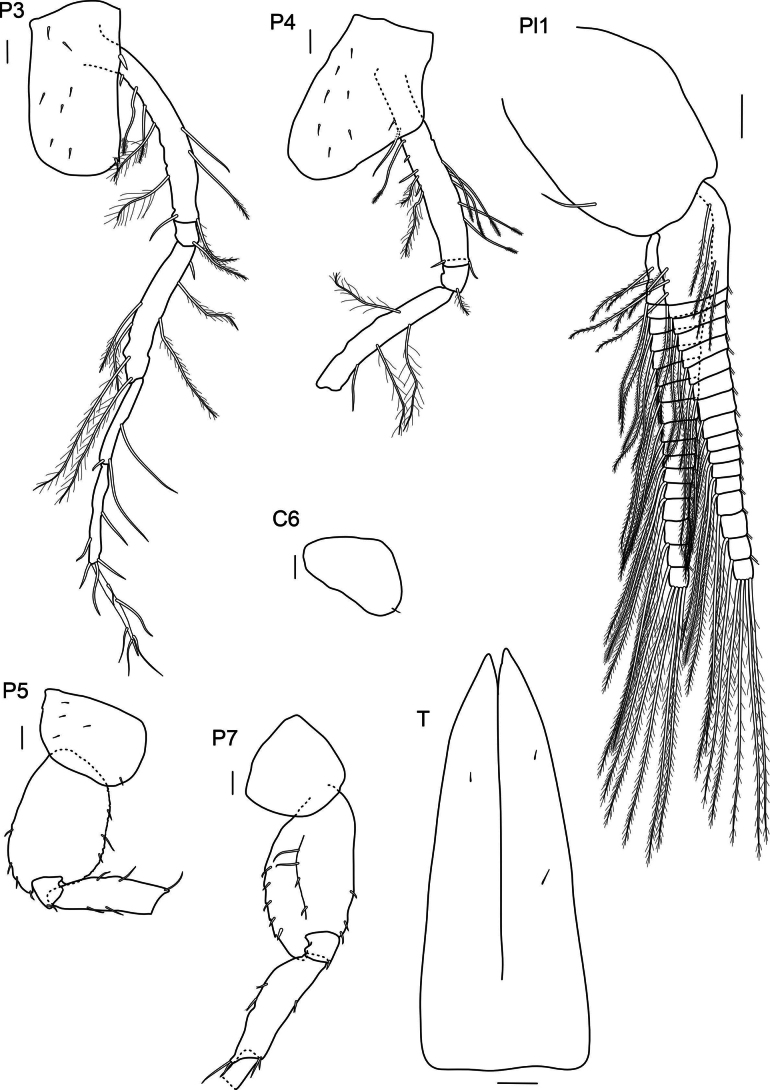
*
Cleonardo daniela* sp. nov., holotype, SMF 62810, male, 6.5 mm, Pacific, for clarity some plumose setae are omitted from the pleopod 1. Scale bars: 0.1 mm.

***Pereopod 3*** (Fig. 4): coxa rectangular, rounded anteriorly, posterior angle with little notch and spine, with strong spine proximally on posterior margin; basis long and elongated; basis, ischium and merus with long plumose setae of different length; carpus same length as propodus but shorter than merus; dactylus same length as propodus and with simple setae.

***Pereopod 4*** (Fig. 4): coxa narrowing distally, rounded, posterior margin weakly excavated, anterior edge nearly straight; basis, ischium and merus similar to pereopod 3; carpus and subsequent articles broken off.

***Pereopod 5*** (Fig. 4): coxa < 1/2 length of coxa 4, rounded, broader than long; basis very broad, margins weakly serrated and with spines, anteroventral lobe small, posteroventral lobe very short; merus shorter than basis; carpus and following articles broken off, but as seen in paratype SMF 62812; propodus longer than carpus, dactylus 3/4 of length of propodus, inner margin of dactylus and propodus with row of small spines.

***Pereopod 6*** (Fig. 4): coxa short, with broadly rounded posterior lobe, posteroventral lobe reaching end of ischium, subsequent articles broken off, but as seen in paratype SMF 62812; basis to propodus similar to pereopod 5.

***Pereopod 7*** (Fig. 4): coxa as long as broad, nearly quadrate but upper margin slightly longer than lower and with rounded corners; basis ovate, with spines on margins and long setae in a midline, posterior margin with five serrations each with inset small spine, posteroventral lobe reaching end of ischium; ischium with one spine at anterior edge; merus same length as basis, with elongated posterior edge, that ends with two spines, shorter anterior edge with two spines; carpus to dactylus broken off, but as seen in paratype SMF 62812; propodus is longer than carpus.

**Pleon: *Epimeral plate 1*** (Fig. 1): anterior and posterior margins rounded, ventral margin convex.

***Epimeral plate 2*** (Fig. 1): anterior margin concave with produced rounded edge, posterior margin rounded with acute corner, ventral margin nearly straight.

***Epimeral plate 3*** (Fig. 1): anterior margin similar to epimeral plate 2, posteroventral corner with small tooth, ventral margin convex.

**Urosome: *Urosomites 1–3*** (Fig. 1): urosomite 1 slightly longer than urosomites 2 and 3 combined; urosomite 2 shortest.

***Uropod 1*** (Fig. 3): lanceolate; peduncle slightly longer than inner ramus, dorsolateral and dorsomedial margins with 12 and five spines, respectively; outer ramus 1/5 shorter than inner ramus, dorsolateral and dorsomedial margins with seven and two spines, respectively; inner ramus nearly reaching outer ramus of uropod 2, dorsolateral and dorsomedial margins with four and five spines, respectively.

***Uropod 2*** (Fig. 3): lanceolate; peduncle same length as outer ramus, dorsolateral and dorsomedial margins with five and two spines, respectively; outer ramus ~ 3/4 length of inner ramus, dorsolateral and dorsomedial margins with five and three spines, respectively.

***Uropod 3*** (Fig. 3): lanceolate; peduncle short, > 1/2 length of inner ramus, with few spines; outer edge forming acute tip; outer ramus 3/4 length of inner ramus, dorsolateral and dorsomedial margins with three and six spines, respectively.

***Telson*** (Fig. 4): deeply cleft, ~ 75%; elongate, narrowing distally, with few spines, lobes nearly acute.

##### Remarks.

*Cleonardo daniela* sp. nov. is most similar to *Cleonardo longipes* Stebbing, 1888 but differs in the following characters: eyes present, antenna 1 shorter than antenna 2, corners of article 1 not produced, but forming two small teeth on lateral side, article 2 forming acute extension on outer lateral side nearly reaching 1/2 of article 3, uni-articulated accessory flagellum broader and nearly reaching 1/2 of first article of flagellum, first article of flagellum shorter than in *Cleonardo longipes*. Morphological differences to *C. longipes* and other closely related species are summarized in Table 3.

**Table 3.** Morphological differences between *Cleonardo daniela* sp. nov., *C. compassionate* sp. nov., *C. longipes* Stebbing, 1888, and *C. biscayensis* Chevreux, 1908.

**Species/character**s	*** C. daniela* sp. nov**.	*** C. compassionate* sp. nov**.	*** C. longipes***	*** C. biscayensis***
Antenna 1: length of article 1 to article 2	equal	shorter	shorter	equal
Accessory flagellum	1/2 the length of first article of flagellum	same length as first article of flagellum	shorter than first article of flagellum	same length as first article of flagellum
Antenna 2: calceoli on articles	posterior edge of article 4, 5 and flagellum	posterior edge of article 4, 5 and flagellum	anterior edge of article 4, 5 and flagellum	anterior edge of flagellum
Mandible: length of article 2 to article 3	art. 2 > art. 3	art. 2 = art. 3	art. 2 < art. 3	art. 2 < art. 3
Mandible: accessory spine row	5 or 6	4 or 5	10 or 11	–
Telson exceeding uropods	no	yes	no	–
Sexual dimorphism	no	yes	no	yes

##### Etymology.

This species is named for Daniela Engel, the mother of the author LE. The name is used as a noun in apposition.

##### Distribution.

Abyssal Pacific Ocean, Clarion-Clipperton Zone in 4170 m – 4209 m depth.

##### Molecular identification.

Following the definition given by Pleijel et al. (2008), the sequence of the holotype male of *Cleonardo daniela* sp. nov. (SMF 62810, GenBank accession number PQ734433) is designed as a hologenophore of all obtained sequences. The COI sequences of the single paratype and additional individuals are also available in GenBank (Table 2). Furthermore, the species has received a Barcode Index Number from Barcode of Life Data Systems: BOLD:AEB2179 (https://doi.org/10.5883/BOLD:AEB2179).

#### Cleonardo compassionate sp. nov.

Taxon classificationAnimaliaAmphipodaEusiridae

74BB3C18-7CA8-59B0-9B59-46A46756B7D9

https://zoobank.org/13C58B3A-50FB-4367-A7E6-71204EF49409

Figs 5, 6, 7, 8, 9

Eusiridae ADF7841: Jażdżewska and Mamos 2019: fig. 2A.

##### Type material.

***Holotype***: Pacific • mature female, 10 mm, SMF 62814, COI: PQ734425, body remnants and one slide with appendages; Clarion-Clipperton Zone, OMS exploration contract area, R/V *Thompson*, ABYSSLINE-2, EBS, AB2-EB06, 01/03/2015, 12°15.06'N, 117°19.2'W, 4137 m. ***Paratype***: Pacific • mature male, 10 mm (urosome broken off), SMF 62815, COI: PQ734442, Clarion-Clipperton Zone, BGR exploration contract area, R/V *Sonne*, MANGAN 2018, EBS, SO 262-150, 07/05/2018, 11°50.009'N, 116°14.780'W–11°49.978'N, 116°13.316'W, 4074–4095 m.

##### Additional material.

Pacific • Clarion-Clipperton Zone; BGR exploration contract area, several stations • abyss adjacent to Kuril-Kamchatka Trench, one station (Table 2).

##### Type locality.

Abyssal Pacific Ocean, Clarion-Clipperton Zone, 12°15.06'N, 117°19.2'W, 4137 m.

##### Description.

**Figure 5.**
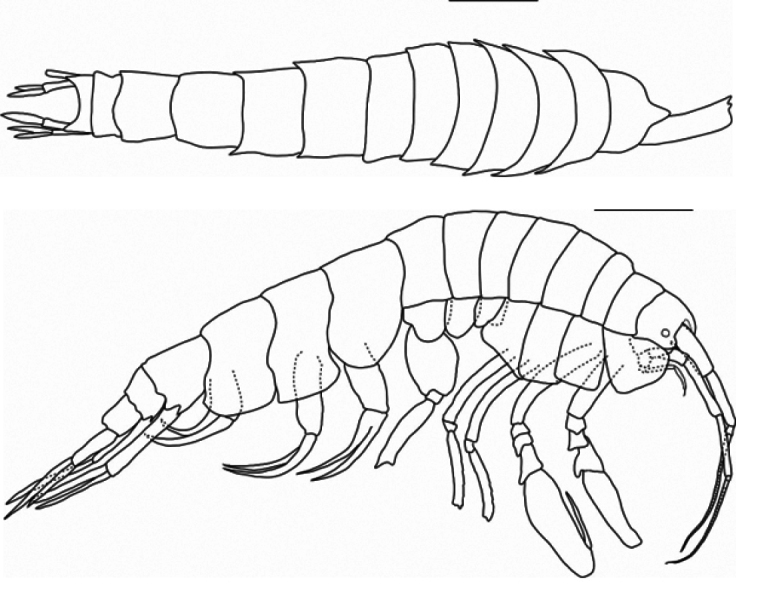
*
Cleonardo compassionate* sp. nov. dorsal and lateral habitus of holotype (female, 10 mm, SMF 62814). Scale bars: 1 mm.

**Figure 6.**
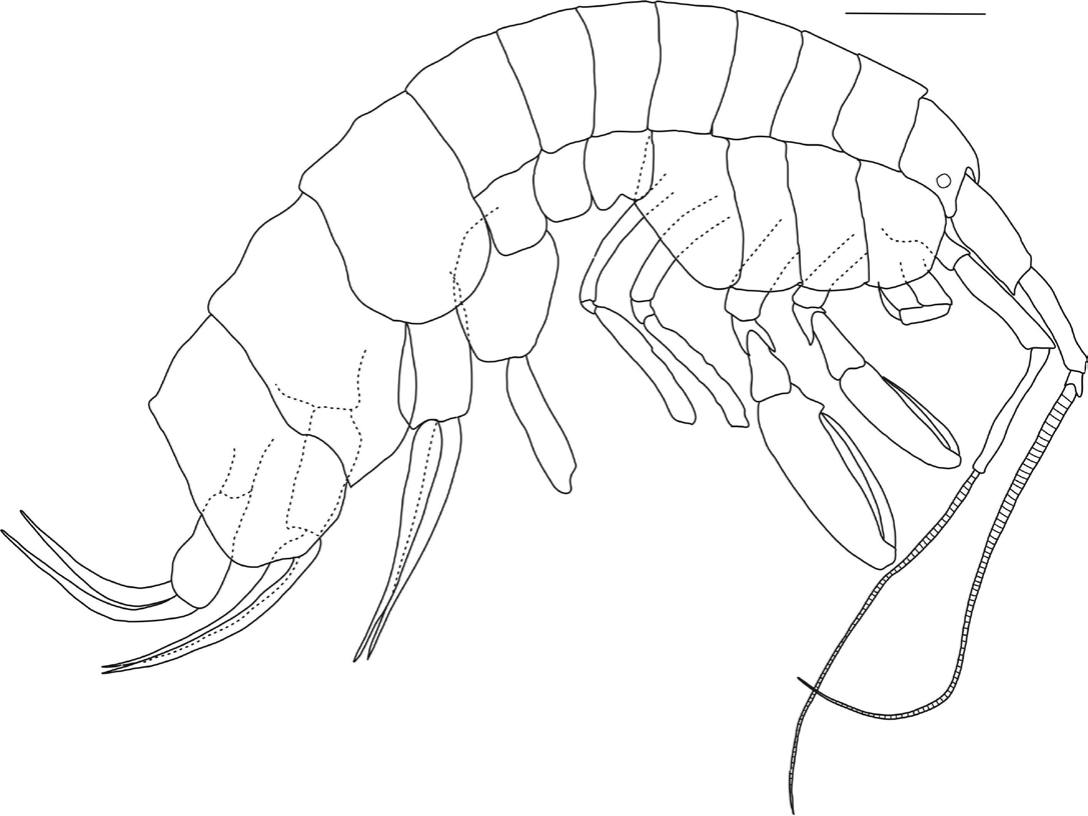
*
Cleonardo compassionate* sp. nov. lateral habitus of paratype (male, 10 mm, SMF 62815). Scale bar: 1 mm.

***Body*** (Figs 5, 6): dorsally smooth.

***Head*** (Figs 5, 6): rostrum slightly curved and 1/3 length of peduncle 1 of antenna 1; head lobes rounded, slightly produced.

***Eyes*** (Figs 5, 6): present, small, rounded, white in preserved specimen.

**Figure 7.**
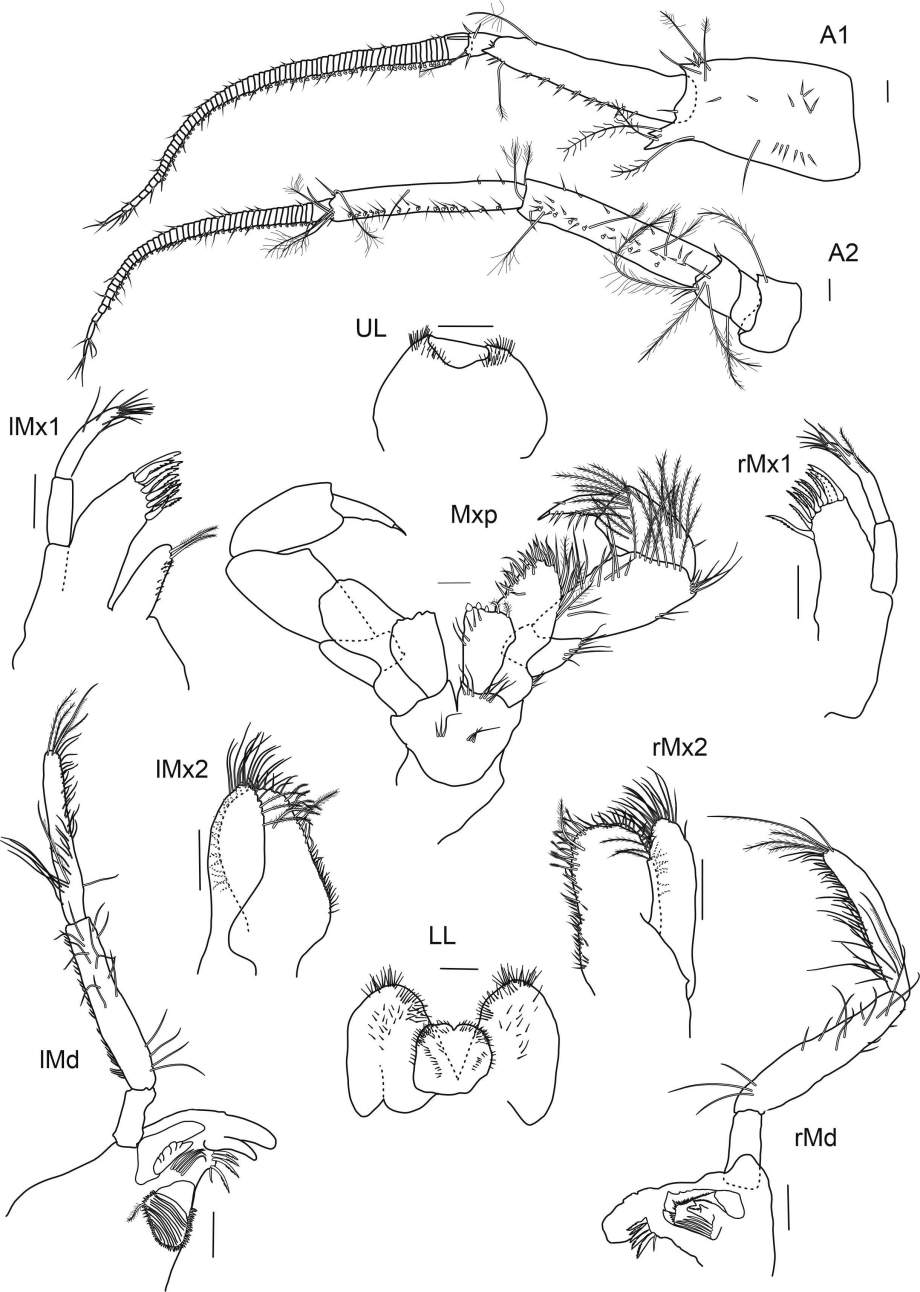
*
Cleonardo compassionate* sp. nov. holotype, female, 10 mm, SMF 62814, Pacific, in maxilliped not all spines and setae are shown for clarity. Scale bars: 0.1 mm.

***Antenna 1*** (Fig. 7): ~ 1/2 body length, same length as antenna 2; article 1 of antenna 1 length 2× as long as broad and extended into a blunt point; article 2 thinner and longer than article 1 and ending in a dentate extension; simple and plumose setae on articles 1 and 2; article 3 short, but longer than first segment of flagellum; accessory flagellum uni-articulated, thin and as long as first article of flagellum; flagellum 69-articulated and longer than articles 1, 2, and 3 combined, articles of flagellum short except for first article which is as long as next four articles, and last six articles which are longer than previous articles, with the last one the longest; calceoli on posterior margin of flagellum, no calceoli on last six segments; short simple setae along flagellum.

***Antenna 2*** (Fig. 7): ~ 1/2 body length; articles 1–3 short, with plumose setae; article 3 length 1/5 of article 4; article 4 broader and slightly longer than article 5, both articles with simple and plumose setae as well as calceoli on posterior margin; flagellum 46-articulated, shorter than articles 4 and 5 combined; articles short except for first article which is as long as next four articles, and last three articles longer than previous ones; calceoli on posterior margin of flagellum, but no calceoli on last four articles; short setae on flagellum.

***Mandibles*** (Fig. 7): palp tri-articulated, article 1 length 1/3 of article 2; article 2 of left mandible with many short setae on outer margin; article 3 little shorter than article 2; article 3 with one strong pectinated setae on lateral side and at apex; articles 2 and 3 with long setae and additionally with few plumose setae on tip of article 3; right lacinia mobilis tridentate, incisor is one long tooth plate; mandible corpus with five dentated setae and one normal thin seta, left lacinia six-dentate, mandible corpus with row of four dentated setae and one thin normal one; molar tubercle of left mandible prominent with dozens of denticles, and carrying a small plumose setae at the lower corner; molar of right mandible missing.

***Maxillae 1*** (Fig. 7): inner lobe of right maxilla 1 missing; inner lobe of left maxilla 1 not reaching length of outer lobe with two plumose setae; outer lobe with 11 spine-teeth, first row with four spine-teeth of which three are six-dentate and one uni-dentate, second row with seven spine-teeth of which six are more than ten-dentate and one uni-dentate.

***Maxillae 2*** (Fig. 7): inner lobe broader and reaching length of outer; inner lobe with short smooth setae and plumose setae, additionally one stronger plumose seta; posterior and lower anterior margins with very thin setae; outer lobe with long simple setae on tip and long plumose ones on inner margin.

***Maxilliped*** (Fig. 7): inner lobes nearly reaching length of article 1 of palp with three teeth and thick plumose setae; outer lobes reaching 1/2 of article 2 of palp, with many strong setae along inner margin, setae on apex and posterior margin longer and plumose; palp four-articulated; article 1 length 1/2 of article 3; articles 2 and 3 with longer plumose setae; dactylus with seven strong setae.

***Upper lip*** (Fig. 7): entire, ventral margin broadly rounded, with fine setae.

***Lower lip*** (Fig. 7): broad, outer lobes with little incision.

**Figure 8.**
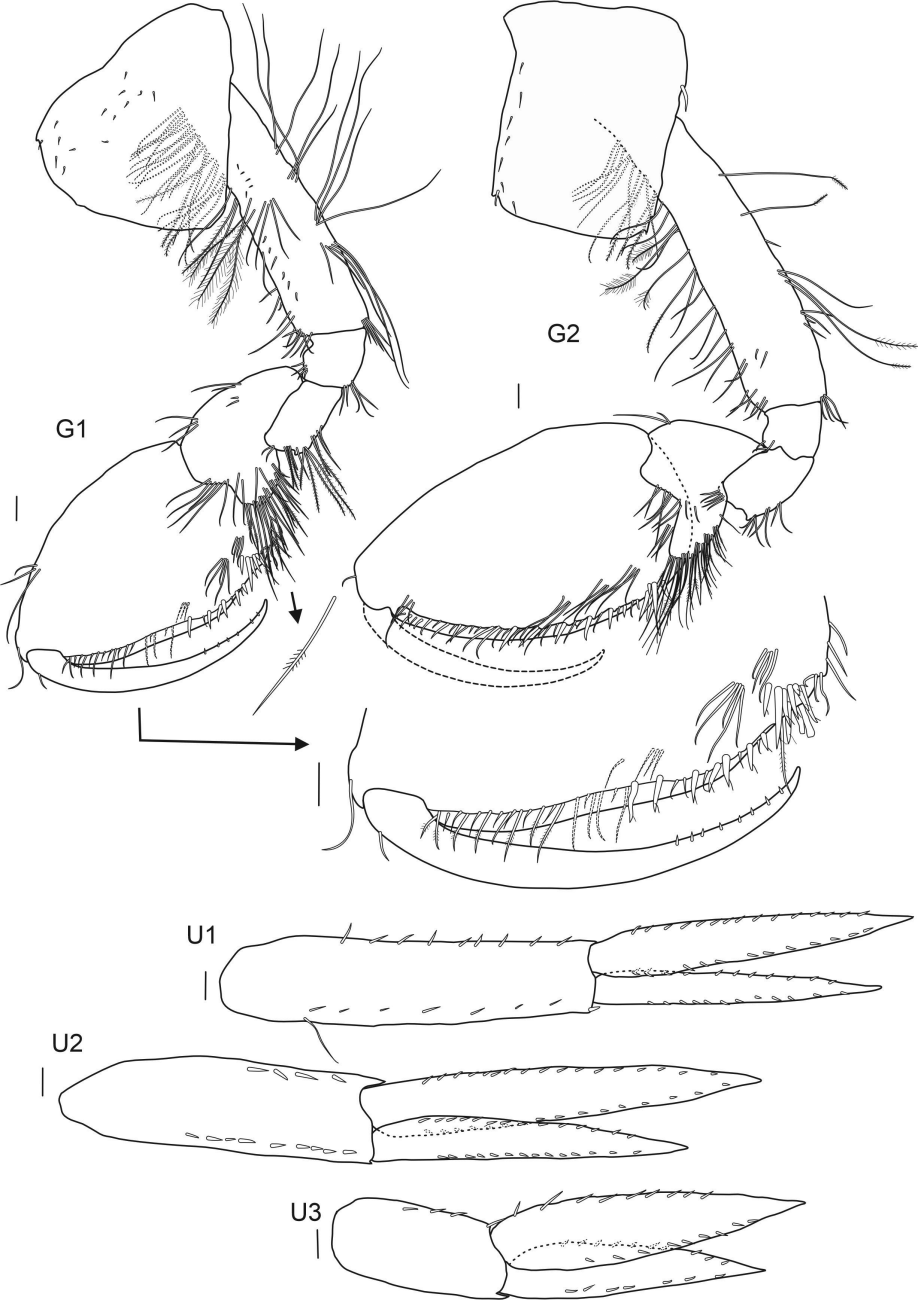
*
Cleonardo compassionate* sp. nov., holotype, female, 10 mm, SMF 62814, Pacific. Scale bars: 0.1 mm.

**Pereon: *Gnathopod 1*** (Fig. 8): slightly smaller than gnathopod 2 but of a similar shape, both strongly subchelate; coxa approx. same size as following coxae, strongly widening distally, axe-shaped, anterior corner rounded, slightly produced, posterior corner rounded, little notch on posterior and anterior corner, surface of coxa with few small spines; basis long and elongated, with many plumose and smooth setae; merus with long plumose setae; carpal lobe broad, slightly produced, but not reaching palmar angle, carpal lobe with eight rows of ~ 8 plumose setae, the first row with only two smooth setae the other rows with eight plumose setae; subovate propodus with few setae on posterior margin and apex; palm with plumose setae, shorter smooth setae and seven spines; palmar angle with nine spines of different sizes (two of these are broken and seem to have been very long like in other *Cleonardo* species); dactylus with eight small spines.

***Gnathopod 2*** (Fig. 8): larger than gnathopod 1; coxa nearly rectangular, anteroproximal corner produced, anterodistal corner rounded, with one notch that carries one thin seta, posterior margin straight with one stronger spine on upper half, posterodistal corner with two notches, each carrying one seta, surface of coxa with small spines; basis longer and a bit wider than in gnathopod 1; carpal lobe is more elongated and nearly reaching palmar angle, with eight rows of setae, with the first row only consisting of one smooth seta, the other seven consisting of eight plumose setae; propodus more elongated and more egg-shaped, palm with seven strong spines and shorter smooth and plumose setae, palmar angle with ten strong spines; dactylus broken off.

**Figure 9.**
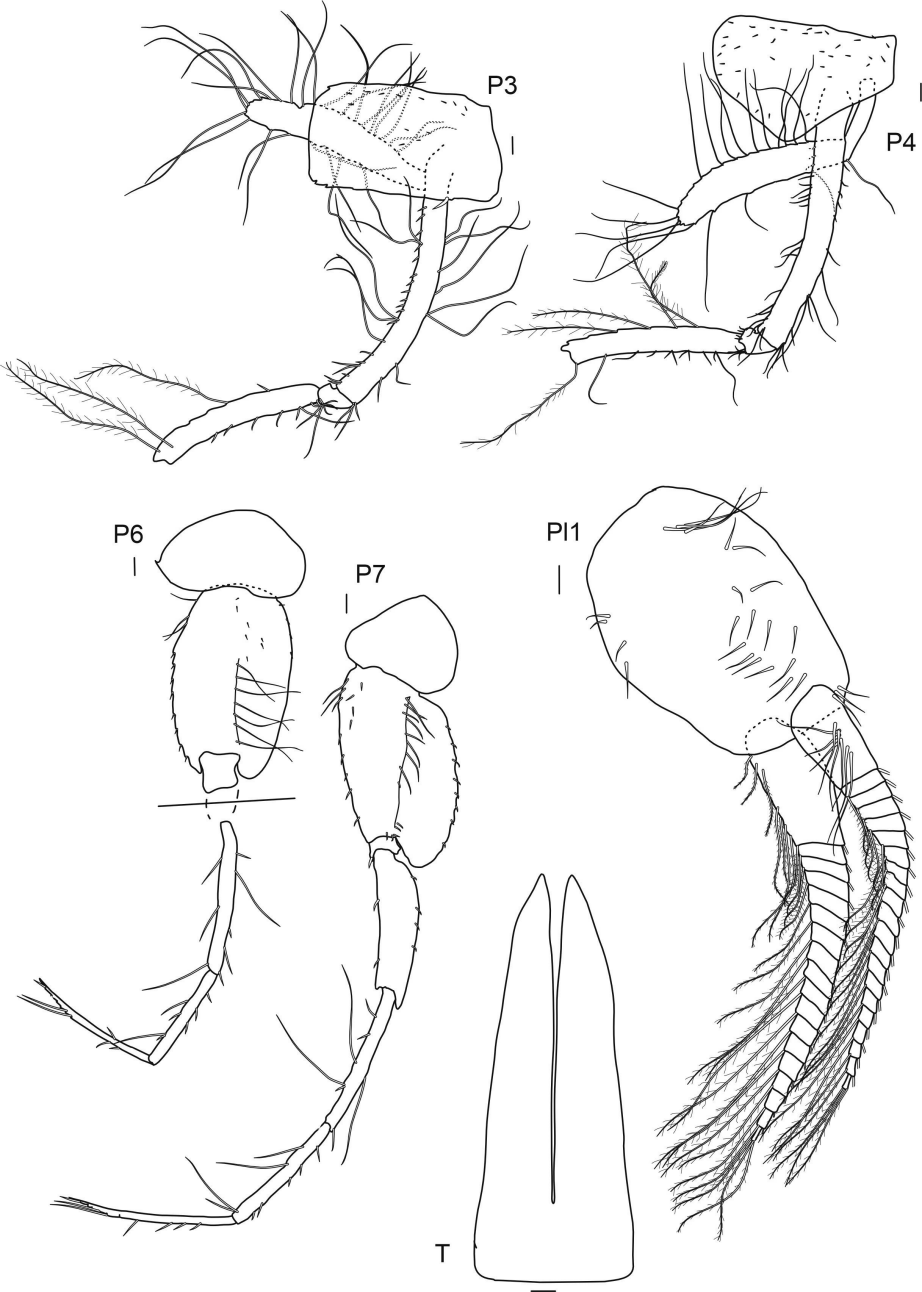
*
Cleonardo compassionate* sp. nov., holotype, female, 10 mm, SMF 62814, Pacific, for pleopod not all setae are shown for clarity. Scale bars: 0.1 mm.

***Pereopod 3*** (Fig. 9): coxa similar to coxa 2, with straight anterior and posterior margins and one little notch at anterodistal corner and two at posterodistal corner, with strong spine proximally on posterior margin, surface of coxa with many small spines; basis long and slender, with short setae on inner, and long setae on outer and inner margins; merus 2/3 length of basis, with long plumose setae and short smooth ones; next articles broken off.

***Pereopod 4*** (Fig. 9): coxa 4 anterior margin straight, rounded below, posterior margin excavated and more pointed, surface of coxa with many small spines; similar to pereopod 3; basis is slender and long, with many smooth and plumose setae; merus shorter than basis but with longer plumose setae; next articles broken off.

***Pereopod 5*** (Figs 5, 6): coxa 5: bilobed, with anterior lobe slightly shorter than posterior lobe; subsequent articles missing.

***Pereopod 6*** (Fig. 9): coxa 2× as broad as long, corners rounded, little notch on anterior margin; basis large and ovate, anterior margin serrated and with spines, surface of basis with spines and row of long setae, posterodistal corner produced along ischium; merus is damaged; carpus longer than propodus; dactylus longer than propodus, with setae along its margins and tip.

***Pereopod 7*** (Fig. 9): coxa anteriorly produced, nearly oval, corners rounded; similar to pereopod 6, but with slightly longer articles; basis large ovate, serrated on posterior margin, with little spines along the margins, surface of basis with row of long setae, posterodistal corner of basis drawn into lobe exceeding ischium; merus shorter than basis, with spines; carpus longer than propodus; dactylus longer than propodus, with setae along its margins and tip.

**Pleon: *Epimeral plate 1*** (Figs 5, 6): anterior and posterior margins rounded, ventral margin convex; plate with small spines.

***Epimeral plate 2*** (Figs 5, 6): anterior margin concave, anterodistal corner rounded and produced, posterior margin convex, posterodistal corner rectangular; plate with small spines.

***Epimeral plate 3*** (Figs 5, 6): anterior margin less concave, anterodistal corner rounded, posterior margin slightly rounded, posterodistal corner slightly acute; plate with small spines.

**Urosome: *Urosomites 1–3*** (Figs 5, 6): urosomite 1 longer than other two.

***Uropod 1*** (Fig. 8): lanceolate; peduncle longer than rami, dorsolateral and dorsomedial margins with eight and nine spines, respectively; inner ramus longer than outer; margins of rami and peduncle with spines on dorsolateral and dorsomedial margins with 11 and 15 spines, respectively; slightly shorter than telson.

***Uropod 2*** (Fig. 8): lanceolate; peduncle as long as outer ramus, with inner corner acute, dorsolateral and dorsomedial margins with four and nine spines, respectively; inner ramus longer than outer; dorsolateral and dorsomedial margins with 14 and 17 spines, respectively; barely exceeding telson.

***Uropod 3*** (Fig. 8): lanceolate; peduncle short, inner corner acute, dorsolateral and dorsomedial margins with four spines; inner ramus longer than outer dorsolateral and dorsomedial margins with eight and nine spines, respectively; barely exceeding telson.

***Telson*** (Fig. 9): deeply cleft, ~ 80%, narrowing distally, lobes slightly separating towards the tip.

***Oostegite*** (Fig. 9): slender and long; serrated and with long setae.

##### Remarks.

*Cleonardo compassionate* sp. nov. shows the most similarity to *Cleonardo daniela* sp. nov., *Cleonardo biscayensis* Chevreux, 1908 and *Cleonardo longipes* Stebbing, 1888, but multiple differences are still present, as summarized in Table 3.

Further differences to *C. longipes* are: eyes present (vs absent); antenna 1, same length as antenna 2 (vs antenna 1 shorter than 2), article 2 with lateral dentate extension (vs no lateral dentation), accessory flagellum thin and as long as first article of flagellum (vs accessory flagellum shorter), flagellum 69-articulated with first article of flagellum as long as the three following articles, articles of flagellum very thin (vs first article as long as five or six subsequent articles and not thin); antenna 2, articles 4 and 5 equal in length and both with calceoli on posterior margin (vs article 4 longer than article 5 and calceoli on anterior margin); mandible, palp article 1 longer, ~ 1/3 of article 2, article 3 shorter than article 2 (vs article 2 shorter); maxilliped, dactylus with 7 short strong setae (vs 10 setae); coxa 1, no spines on hind margin (vs one spine on hind margin); gnathopod 1, carpal lobe rounded and weakly produced (vs carpal lobe more elongated and nearly reaching palmar angle); gnathopod 2, carpal lobe more elongated than in gnathopod 1, but still quite short (vs carpal lobe similar as in gnathopod 1); pereopods 5–7, carpus longer than propodus, dactylus same length as propodus (vs dactylus 2/3 of length of propodus and carpus shorter than propodus); uropod 3 without plumose setae (vs with plumose setae). The differences between the new species and *C. biscayensis* are as follows: eyes present (vs absent); antenna 1, same length as antenna 2 (vs antenna 1 longer than 2); antenna 2 with calceoli on posterior margin (vs calceoli on anterior margin), flagellum shorter than articles 4 and 5 combined (vs flagellum much longer); mandible, palp article 1 longer, ~ 1/3 of article 2, article 3 shorter than article 2 (vs article 2 shorter than article 3); gnathopod 1, carpal lobe rounded and weakly produced (vs carpal lobe more elongated and nearly reaching palmar angle); pereopods 5–7, carpus longer than propodus, dactylus same length as propodus (vs carpus not longer than propodus); uropod 3 with both margins of both rami with multiple spines (vs uropod 3 with one additional strong spine on inner ramus).

##### Variation.

This species shows sexual dimorphism. Antennae 1 and 2 in males are longer than in females, article 1 of antenna 1 has long rows of brush setae (vs no brush setae in female). Additionally, males have calceoli on flagellum (Figs 11, 12).

**Figure 10.**
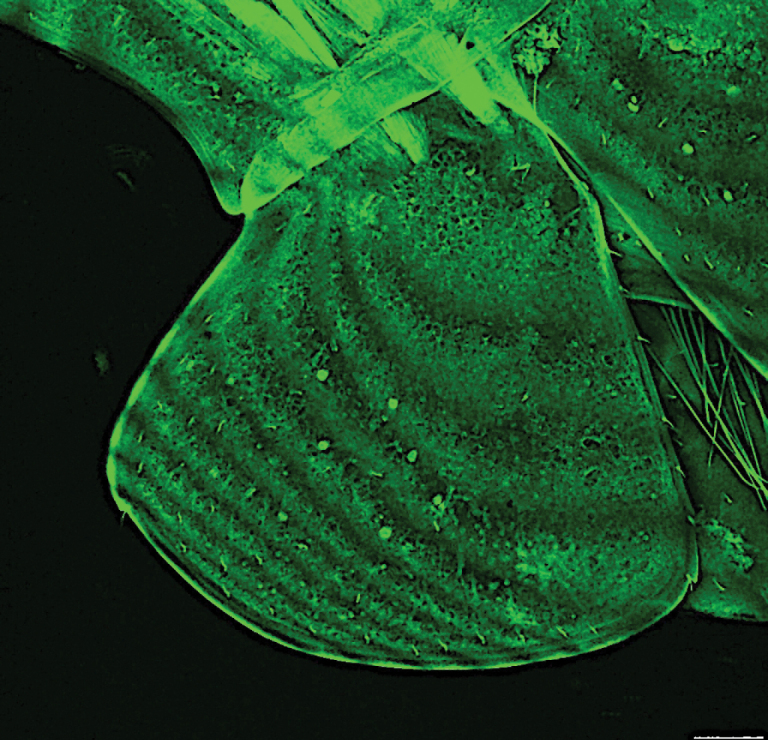
*
Cleonardo compassionate* sp. nov., holotype, SMF 62814, coxa 1. Scale bar: 100 µm.

**Figure 11.**
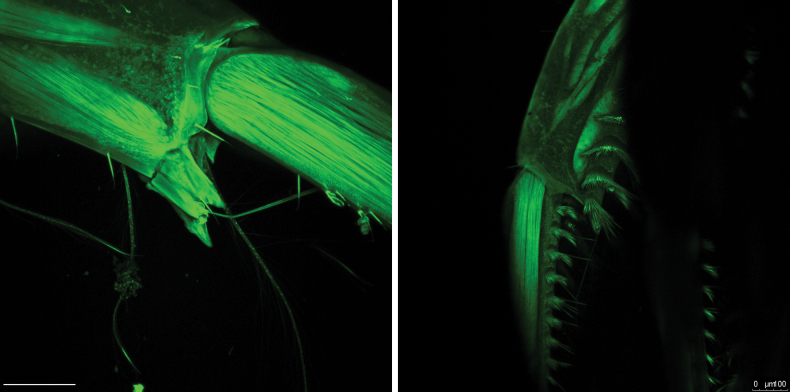
CLSM image of bidentate extension of article 1 of antenna 1 in *Cleonardo compassionate* sp. nov.: left image, holotype SMF 62814, female without brush setae and broken extension of article 1; right image, paratype SMF 62815, male with brush setae. Scale bars: 75 µm (left image); 100 µm (right image).

**Figure 12.**
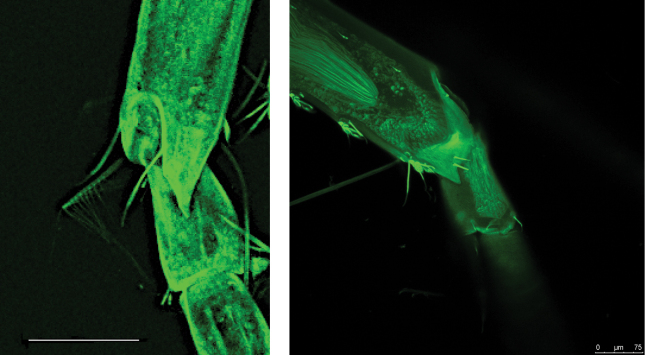
CLSM image of dentate extension of article 2 of antenna 1 in *Cleonardo daniela* sp. nov. left image, holotype SMF 62810, male; and *Cleonardo compassionate* sp. nov. right image, holotype SMF 62814, female. Scale bars: 100 µm (left image); 75 µm (right image).

##### Etymology.

The name ‘*compassionate*’ is used as a noun in apposition, defined as the feeling that arises when you are confronted with another’s suffering and feel motivated to relieve that suffering.

##### Distribution.

Abyssal Pacific Ocean, Clarion-Clipperton Zone in 4074–4352 m depth and Northwest Pacific Ocean, Kuril-Kamchatka Trench area in 5222–5223 m.

##### Molecular identification.

Following the definition given by Pleijel et al. (2008), the sequence of the holotype male of *Cleonardo compassionate* sp. nov. (SMF 62814, GenBank accession number PQ734425) is designed as a hologenophore of all obtained sequences. The sequences of the paratypes and additional individuals of the species are also available in GenBank (Table 2). Furthermore, the species has received a Barcode Index Number from Barcode of Life Data Systems: BOLD:ADF7841 (https://doi.org/10.5883/BOLD:ADF7841).

#### Genus Dorotea

Taxon classificationAnimaliaAmphipodaEusiridae

Corbari, Frutos & Sorbe, 2019

85D274B7-9E80-5028-B451-AF128F3FA8AF

##### Type species.

*Dorotea papuana* Corbari, Frutos & Sorbe, 2019 (original designation).

##### Included species.

*Dorotea aberrantis* (Bellan-Santini & Ledoyer, 1987); *D. papuana* Corbari, Frutos & Sorbe, 2019; *Dorotea elizae* sp. nov.

##### Diagnosis.

(updated after Corbari et al. 2019). Body not strongly compressed, dorsally smooth. Rostrum short. Head lateral cephalic lobe weakly produced. Eyes lacking. Antennae medium size. Antenna 1 probably slightly longer than antenna 2, peduncular article 1 subequal to article 2, accessory flagellum uni-articulated, linear. Antenna 2, peduncular article 4 broader than article 5, flagellum not shortened. Upper lip evenly rounded distally. Lower lip, inner lobes weak. Mandible, incisor with long incurved cutting edge, poorly dentate or smooth; lacinia mobilis five-dentate; setal row with six or seven setae; palp, article 3 longer than article 2, slightly falciform, tapering distally; article 2 broader than article 3. Maxilla 1, inner plate with 1–3 subapical setae; outer plate with ten or 11 apical stout setae; palp slender, distal article longest. Maxilla 2, inner plate broader and shorter than outer plate. Maxilliped palp, articles ordinary; outer plate large; inner plate with three apical teeth. Coxae 1–4 regular, medium; coxa 1 broadly rounding, not produced; coxa 4 slightly excavate posteriorly. Coxae 5 and 6 bilobate. Gnathopods 1 and 2 subsimilar, both strongly subchelate; carpal lobes broad; propodus large, posterior margin short, palm oblique, margin spinose. Pereopods 3 and 4 slender, merus slightly longer than carpus, dactylus simple, stout and medium length. Pereopods 5–7 homopodous, subequal in form and size, not greatly elongate; bases broad; dactylus simple, stout and medium length. Epimera 1–3 regular, not serrate on posterior margin. Uropods 1 and 2 rami lanceolate, outer ramus shorter than inner ramus. Uropod 3 rami broadly lanceolate, outer ramus shorter than inner ramus. Telson elongate, lobate, weakly cleft. Brood plates on pereopods 2–5. Coxal gills on pereopods 2–7.

The genus *Dorotea* was created for *Dorotea papuana* Corbari, Frutos & Sorbe, 2019 and also includes *D. aberrantis* (Bellan-Santini & Ledoyer, 1987) previously assigned to *Eusiroides* (Bellan-Santini and Ledoyer 1987). Our individual matches the generic diagnosis in most characters. However, seven characters appear to vary between species assigned to this genus (see Table 4).

**Table 4.** Characters used to separate species of *Dorotea*. Character states listed in the genus diagnosis indicated with bold font.

**Species/ character**	*** Dorotea aberrantis* (Bellan-Santini & Ledoyer, 1987)**	*** Dorotea papuana* Corbari, Frutos & Sorbe, 2019**	*** Dorotea elizae* sp. nov**.
A1/A2 calceoli	unknown in female, absent in male	**present in female, unknown in male**	unknown in female, present in male
Upper lip	**rounded distally**	**rounded distally**	slightly emarginate distally
Md cutting edge	smooth	**poorly dentate (2+)**	smooth
Md setal row (no of setae)	seven	**six**	seven
Mx1 inner plate (no of setae)	one	**three**	two
Mx1 outer plate (no of setae)	unknown	**ten**	eleven
Basis P5-7	**with posterodistal lobe**	**with posterodistal lobe**	without posterodistal lobe

Each of the species of *Dorotea* is known only from a unique individual of a different sex (*D. papuana* from the female, for the other two species only males are known), so there is no possibility to infer the intraspecific variation. With the exception of the shape of the upper lip these characters need to be treated as species specific ones. There is a large difference in shape of the upper lip between our specimen and the other two *Dorotea* species described; however, due to the three-dimensional nature of this appendage the differences in observed shape may be associated with positioning during slide preparation. The lack of more individuals of any of the species prevents from decision how stable is this character; this is why the shape of the upper lip was left unchanged in the generic diagnosis.

#### Dorotea elizae sp. nov.

Taxon classificationAnimaliaAmphipodaEusiridae

2B8536E9-8431-5136-BA6B-274294C6F72A

https://zoobank.org/A15B00BE-8C92-4811-AEA1-25F7BF6F4C53

Figs 13, 14, 15, 16

##### Type material.

***Holotype***: Pacific • male, 16 mm, SMF 62816, COI: PQ734718, body remnants and two slides with appendages, Clarion-Clipperton Zone, OMS exploration contract area, R/V *Thompson*, ABYSSLINE-2, EBS, AB2-EB06, 01/03/2015, 12°15.06'N, 117°19.2'W, 4137 m.

##### Type locality.

Abyssal Pacific Ocean, Clarion-Clipperton Zone, 12°15.06'N, 117°19.2'W, 4137 m.

##### Description.

Based on male, 16 mm, SMF 62816.

**Figure 13.**
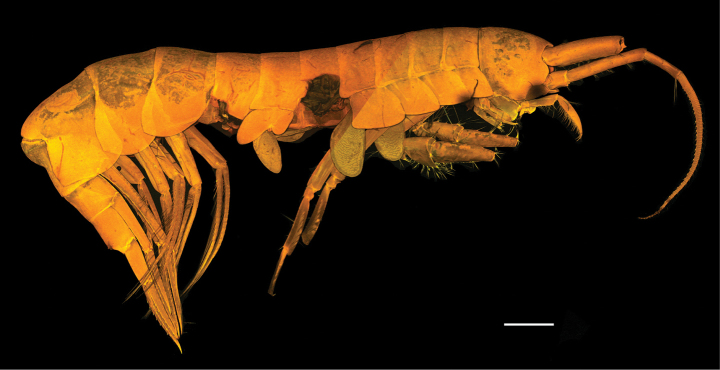
*
Dorotea elizae* sp. nov., male, 16 mm, SMF 62816. Scale bar: 0.5 mm.

***Body*** (Fig. 13): dorsally smooth.

***Head*** (Fig. 13): almost as long as deep, as long as pereonites 1 and 2 combined; no eyes or ocular pigment visible; rostrum vestigial; interantennal lobe weak, subquadrate.

**Figure 14.**
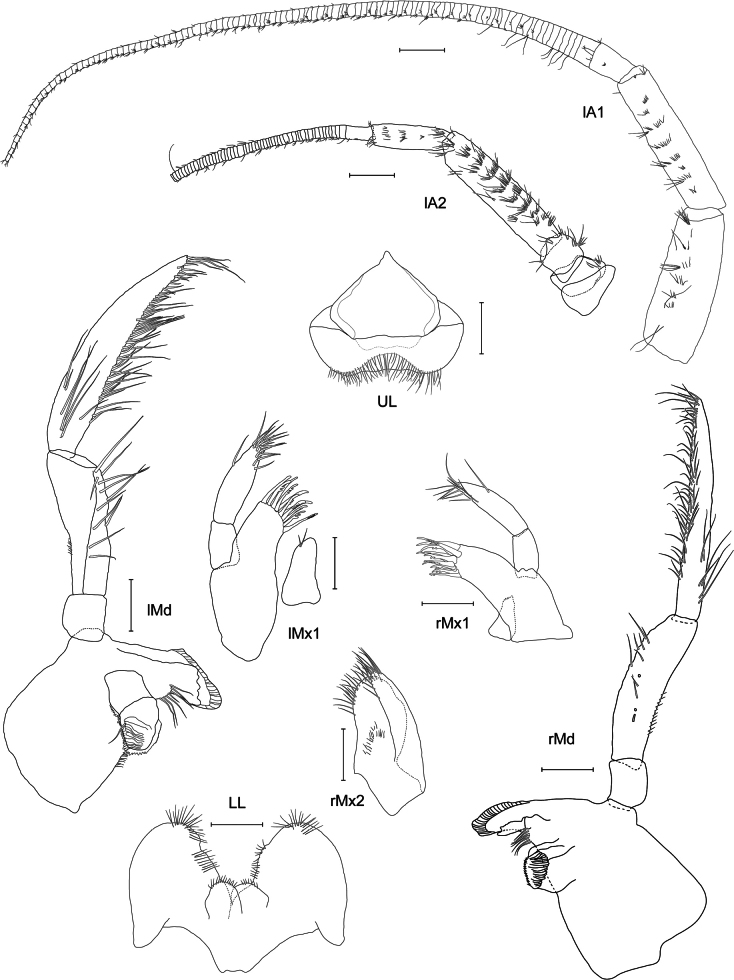
*
Dorotea elizae* sp. nov., Holotype, male, 16 mm, SMF 62816. Scale bars: 0.5 mm (A1, A2), 0.2 mm (LL, lMd, rMd, lMx1, rMx1, lMx2, rMx2, UL).

***Antenna 1*** (Fig. 14): long, as long as pereon and pleon segments combined (checked before dissection), length ratios of peduncle articles 1–3 1:1:0.3; article 1 with seven groups of slender setae and a few additional displaced long setae; article 2 with eight rows of slender setae and additional setae at the distal margin; article 3 with one group of short, slender setae and a few setae at the distal margin; flagellum ≥ 100-articulated, a few distal ones broken off, almost every flagellar article with a few long, slender setae on ventral side, additional group of medial setae every few articles, calceoli present; accessory flagellum uni-articulated, reaching 2/3 of the first flagellar article, with three short distal setae.

***Antenna 2*** (Figs 13, 14): shorter than antenna 1, as long as head and pereon segments combined; article 4 length 2× article 5; peduncular article 4 with nine rows of brush setae and at the distal margin; peduncle article 5 with four rows of short setae and group of long setae at the distal margin; flagellum longer than peduncle, ≥ 50-articulated (distal part broken off), short setae placed distally on flagellar articles, calceoli present.

***Upper lip*** (Fig. 14): entire, wider than long, apically emarginate, with submarginal row of setules. Epistome bluntly pointed.

***Mandible*** (Fig. 14): incisor margins curved, with smooth cutting edge; left lacinia mobilis five-dentate; right lacinia mobilis narrower with indistinct teeth; accessory spine rows with seven narrow spines; molar columnar, strongly triturative, denticulate, one associated seta at left molar (at right molar probably broken off); palp tri-articulated, article 1 short, article 2 length 0.6× article 3, with 11 posterodistal setae, article 3 slightly tapering and curved, seven (left) or eight (right) long setae on medial surface proximally, posterior margin with a row of ~ 70 setae of different lengths.

***Lower lip*** (Fig. 14): outer lobes broadly rounded, mandibular lobes bluntly pointed; inner lobes small, separate; inner and outer lobes setulose apically.

***Maxilla 1*** (Fig. 14): inner plate subtriangular (partly damaged at right Mx), with two tiny, subapical setae; outer plate with 11 spine-teeth; palp bi-articulated, longer than outer plate, slender, rounded apically, article 1 length 0.5× article 2, article 2 with 12 (right) or 14 (left) apical/subapical setae in two rows and one lateral seta.

***Maxilla 2*** (Fig. 14): left – lost, right – plates subequal in length, inner plate 2× as wide as outer, with 19 setae apically and subapically organized in two rows, fine setules on the surface; outer plate rounded with ~ 15 apical setae.

**Figure 15.**
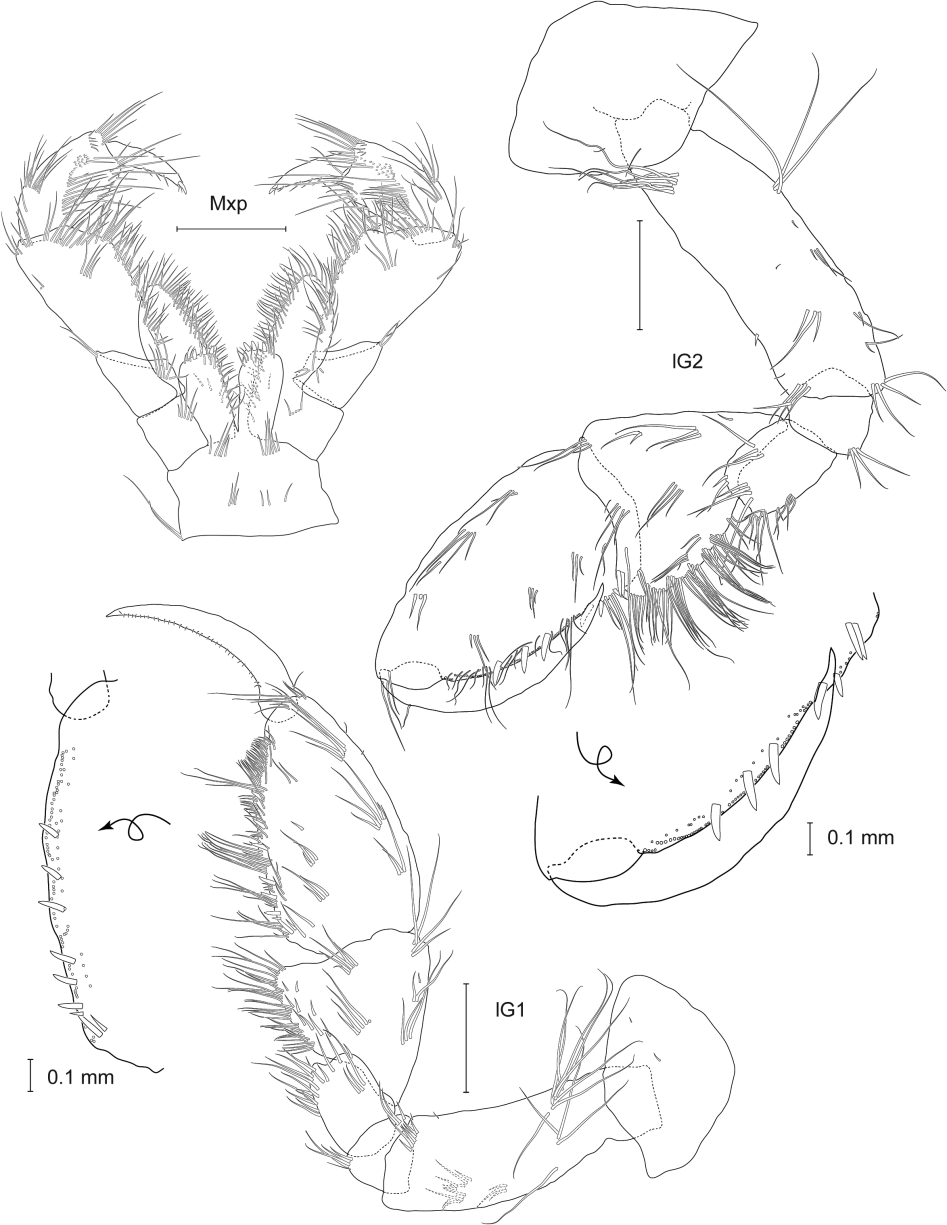
*
Dorotea elizae* sp. nov., Holotype, male, 16 mm, SMF 62816. Scale bars: 0.5 mm if not stated otherwise.

***Maxilliped*** (Fig. 15): strongly setose; inner plate subrectangular, reaching ~ 1/2 basal article of palp, apical margin with three blunt spines and eight longer setae; outer plate slightly curved, long, reaching ~ 1/2 length of palp article 2; palp four-articulated, strong; article 2 widening distally; article 3 expanded mediodistally, with long setae at the edge and on the surface; article 4 strong, slightly curved, with spine on its tip and four seta-like spines; length ratios of articles 1–4 1:2.4:1.8:1.

**Pereon: *Pereonites 1–5*** (Fig. 13) of similar length, pereonites 6 and 7 slightly longer.

***Gnathopod 1*** (Fig. 15): coxa subquadrate, slightly narrowing posteriorly; basis straight, slightly broadening distally, with a group of long setae at the surface at 1/3 of its length, two rows of medium length setae posteriorly at the distal 1/2 of the article and a group of setae at the distal margin; merus posterodistally with 15 setae; carpus expanded, subtriangular, anterior margin with three groups of setae, posterior lobe rounded with setae organized in six rows and a few displaced setae, two rows of five setae each on the surface; propodus subchelate, anterior margin with three groups of long setae, palm oblique, strongly setose with six large and one smaller spines along the margin and additional two at dactylus insertion; dactylus curved, as long as palm with a row of short setules along posterior margin.

***Gnathopod 2*** (Fig. 15): coxa subquadrate, apex rounded, ventral margin naked; basis straight, a group of long setae at the surface at in proximal part, posterior margin with irregularly placed setae of different length, a group of three medium length setae at anterodistal corner and of four setae at posterodistal corner; merus, posterodistally with 18 setae, additional group of three setae on the medial surface; carpus expanded, subtriangular, anterior margin with three groups of setae, posterior lobe rounded with setae organized in seven rows, two rows of four setae each on the surface; propodus subchelate, anterior margin with four groups of long setae, palm oblique, strongly setose with four large and one small spines along the margin and additional two below dactylus insertion; dactylus curved, slightly shorter than palm.

**Figure 16.**
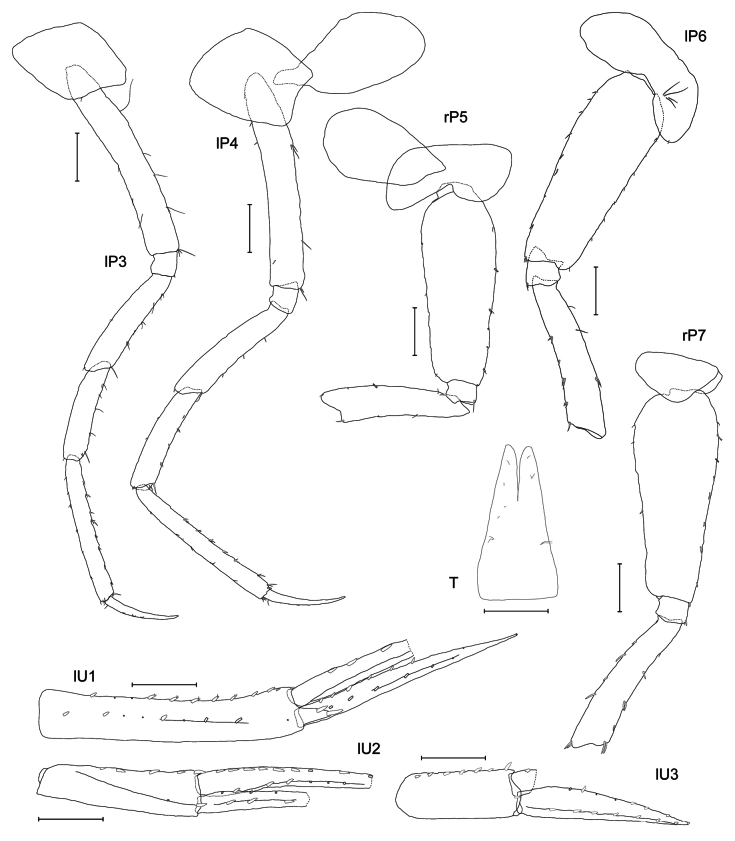
*
Dorotea elizae* sp. nov., Holotype, male, 16 mm, SMF 62816. Scale bars: 0.5 mm.

***Pereopod 3*** (Fig. 16): coxa subrectangular, ventral margin naked; basis long and narrow, length 6.9×width, three setae along anterior margin, posterior margin with four medium length setae, two short and one medium length setae at posterodistal corner; merus slightly expanded distally, short setae in four groups along posterior margin, one longer seta at posterodistal corner; carpus, posteriorly armed with short setae irregularly placed; propodus posterior margin with eight groups of short setae and spines, one at the posterodistal corner; dactylus thin, slightly curved, with three setae along anterior margin; length ratio of articles 2–7: 1:0.1:0.55:0.47:0.7:0.35.

***Pereopod 4*** (Fig. 16): coxa wider than deep, with a small posterodistal lobe; remaining articles similar to pereopod 3 except length ratio of articles 2–7: 1:0.1:0.57:0.5:0.7:0.38.

***Pereopod 5*** (Fig. 16): coxa bilobed with weak posterior excavation, posterior lobe deeper than anterior lobe; basis weakly expanded, without posterodistal lobe, length 3× width, traces of six stouter setae along anterior margin, trace of one seta at anterodistal corner, posterior margin slightly crenulated; merus length 0.6× basis, with traces of two setae along anterior margin; carpus–dactylus broken off.

***Pereopod 6*** (Figs 13, 16): coxa bilobed, anterior lobe significantly smaller than long posterior lobe; weakly expanded, without posterodistal lobe, length 3× width, nine short setae along anterior margin, one short seta at anterodistal corner, posterior margin slightly crenulated with short setules in the crenulations; merus length 0.7× basis, anterior margin with short spines in four groups, some setae along posterior margin; carpus–dactylus broken off.

***Pereopod 7*** (Fig. 16): coxa small, subtriangular; basis weakly expanded anteriorly and tapering distally, without posterodistal lobe, length 2.8× width, anterior margin with seven short setae, posterior margin slightly crenulated; merus length 0.7× basis, with some setae and small spines along anterior and posterior margins, groups of two spines at both anterodistal and posterodistal corners; carpus–dactylus broken off.

**Pleon: *Pleonites 1–3*** (Fig. 13): dorsally smooth.

***Epimeral plates 1–3*** (Fig. 13): anteriorly evenly rounded, posteriorly subquadrate.

***Pleopods*** (Fig. 13): powerful, peduncles and rami long.

**Urosome: *Urosomites 1–3*** (Fig. 13): dorsally smooth, urosomite 1 as long as urosomites 2 and 3 combined, the latter of the same length.

***Uropod 1*** (Fig. 16): lanceolate; peduncle subequal in length to inner ramus, dorsolateral and dorsomedial margins with nine and 11 spines, respectively, blunt distoventral process bearing a distal spine; rami of similar length, outer ramus slightly shorter than peduncle, dorsolateral and dorsomedial margins with six and eight spines, respectively.

***Uropod 2*** (Fig. 16): lanceolate; shorter than uropod 1, peduncle length 0.75× inner ramus, dorsolateral and dorsomedial margins with two and five spines, respectively; inner ramus 1.2× length of outer ramus, both rami bearing dorsolateral and dorsomedial spines.

***Uropod 3*** (Figs 13, 16): lanceolate; peduncle short, length 0.5× inner ramus, dorsolateral and dorsomedial margins with none and seven spines, respectively; outer ramus probably slightly shorter than inner ramus (left: outer ramus broken at 1/5, right: outer ramus broken just before the tip; Fig. 13), dorsolateral and dorsomedial margins both with seven spines.

***Telson*** (Fig. 16): narrowing distally, longer than peduncle of uropod 3, reaching 1/5 of inner ramus (checked before dissection), length 2.75× width, cleft almost 40%, lobes apically rounded but tips slightly damaged, a few setules on dorsal surface.

##### Variation.

The individual is unique so no intraspecific variation may be provided.

##### Remarks.

Apart from the morphological differences between already described species and *D. elizae* sp. nov. listed in Table 4, the new species differs from them in the length of dactyli of pereopods 3 and 4, the shape of bases of pereopods 5–7, the length of rami of uropod 3, and the size of cleft of the telson (Table 5). The recently published article by Weston et al. (2024) provided description of a new genus *Dulcibella* Weston & González in Weston, González, Escribano & Ulloa, 2024 closely related to the *Dorotea*. The morphological description of the new species of this genus, *Dulcibella camanchaca* (Weston & González in Weston, González, Escribano and Ulloa 2024), was supplemented by the COI sequence. These authors mention in their work a “non-public genetic record on BOLD from the Clarion-Clipperton Zone being ~ 90% similar” to their species (Weston et al. 2024). Indeed, the cited record is of *Dorotea elizae* sp. nov. and the p-distance reaches 0.095; however, generic placement of *D. camanchaca* is far from clear and warrants further study. Additional work may result in the synonymisation of the new genus with *Dorotea* as the characters used for its separation seem rather species-specific than being valid at the genus level. Also, the molecular study of Weston et al. (2024) does not fully support *Dulcibella* genus erection. Such work requires supplementary analyses and is beyond the scope of this paper. For this reason we retain the genus allocation as *Dorotea* herein. Due to the fact that the barcode of *D. papuana* was provided (Corbari et al. 2019) it was possible to also make a molecular comparison between that species and *D. elizae* sp. nov. The two available barcodes present 0.155 p-distance that strongly exceeds the threshold used to molecularly delimit amphipod species (e.g., Tempestini et al. 2018). Unfortunately, the remaining species *D. aberrantis* has not yet been barcoded. The present finding of *Dorotea elizae* sp. nov. extends the known geographic distribution of the genus to central East Pacific and also its deepest record to 4137 m.

**Table 5.** Morphological comparison of *Dorotea elizae* sp. nov. and known species with information about their geographic and bathymetric distribution.

**Species/ character**	*** Dorotea aberrantis* (Bellan-Santini & Ledoyer, 1987)**	*** Dorotea papuana* Corbari, Frutos & Sorbe, 2019**	*** Dorotea elizae* sp. nov**.
Outer plate of Mxp	no facial spines	four facial spines	no facial spines
Dactyli P3-4	1/3 length of propodus	1/3 length of propodus	1/2 length of propodus
Basis P5-7 length to width ratio	length 2× width	length 2× width	length 3× width
Basis P5-7 posterodistal lobe	present	present	absent
Telson length to width ratio	length 2.2× width	length 1.9× width	length 2.75× width
Telson cleft	cleft ~ 20%	cleft ~ 20%	cleft almost 40%
Telson armature	two pairs of setae	no setae	a few setules on dorsal surface
Distribution	Indian Ocean, Prince Edward Islands	SW Pacifc, off Budibudi Island, north of Laughlan Archipelago, Solomon Sea	Central E Pacific, Clarion-Clipperton Zone
Depth range	180–232 m	593 m	4137 m

##### Etymology.

The species is named for Eliza Kordula, the girlfriend of the author NĆ.

##### Distribution.

Known only from type locality, Abyssal Pacific Ocean, Clarion-Clipperton Zone, 4137 m.

##### Molecular identification.

Following the definition given by Pleijel et al. (2008), the sequence of the holotype male of *Dorotea elizae* sp. nov. (SMF 62816, GenBank accession number PQ734718) is designed as a hologenophore of all obtained sequences. The species has also received the Barcode Index Number from Barcode of Life Data Systems: BOLD:AEB4129 (https://doi.org/10.5883/BOLD:AEB4129).

#### Genus Rhachotropis

Taxon classificationAnimaliaAmphipodaEusiridae

S.I. Smith, 1883

AF3BC43C-CE82-556E-98B8-9C587E39B69B

Rhachotropis S.I. Smith, 1883: 222. – Stebbing 1906: 847. – Shoemaker 1930: 317. – Gurjanova, 1951:706. – J.L. Barnard 1969: 229. – Barnard and Karaman 1991: 337, figs 59F, 61H, 62B, 63B.
Gracilipes Holmes, 1908: 526.

##### Type species.

*Oniscus aculeata* Lepechin, 1780.

#### Rhachotropis clarionclippertoni sp. nov.

Taxon classificationAnimaliaAmphipodaEusiridae

B898A958-174F-5CF1-A02C-0BAC388411D2

https://zoobank.org/3F2D2E4C-CBEE-4FF2-8FC5-6805B2E0A72F

Figs 17, 18, 19, 20

##### Type material.

***Holotype***: Pacific • female, 8 mm, SMF 62817, COI: PQ734529, body remnants and two slides with appendages, Clarion-Clipperton Zone, OMS exploration contract area, R/V *Thompson*, ABYSSLINE-2, EBS, AB2-EB11, 14/03/2015, 12°2.28'N, 117°14.22'W, 4097 m. ***Paratype***: Pacific • female, 8.5 mm, SMF 62818, COI: PQ734352, Clarion-Clipperton Zone, BGR exploration contract area, R/V *Kilo Moana*, MANGAN 2016, EBS, Ma 16-91, 09/05/2016, 11°49.792'N, 117°30.458'W–11°49.842'N, 117°29.208'W, 4344–4344 m.

##### Additional material.

Pacific • Clarion-Clipperton Zone; BGR exploration contract area, several stations (Table 2).

##### Type locality.

Abyssal Pacific Ocean, Clarion-Clipperton Zone, 12°2.28'N, 117°14.22'W, 4097 m.

##### Description.

Based on holotype, female, 8 mm, SMF 62817.

**Figure 17.**
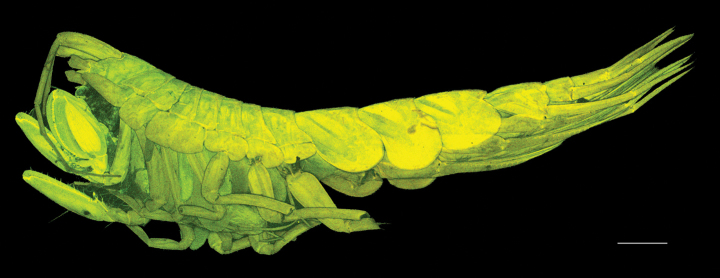
*
Rhachotropis clarionclippertoni* sp. nov., paratype, mature female, 8.5 mm, SMF 62818. Scale bar: 0.5 mm.

**Figure 18.**
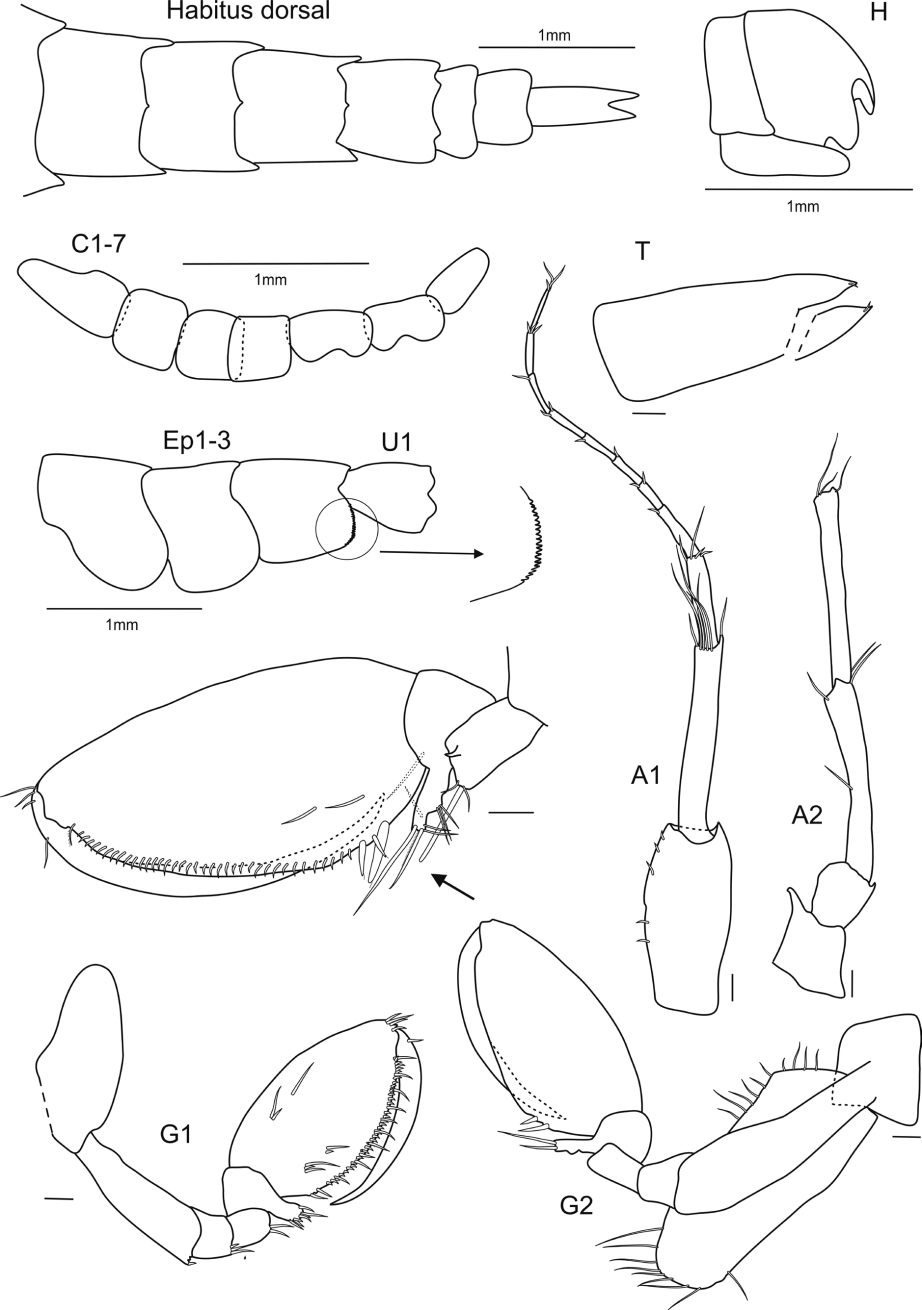
*
Rhachotropis clarionclippertoni* sp. nov., holotype, female, 8 mm, SMF 62817 (H, A1-2, G1-2, T); Paratype, female, 8.5 mm, SMF 62818 (Habitus, C1-7, Ep1-3, U1). Scale bars: 1 mm (habitus, H, C1-7, Ep1-3, U1); 0.1 mm (all remaining appendages).

***Body*** (Figs 17, 18): all pereonites smooth; pleonites 1 and 2 smooth, pleonite 3 with a minute dorsal spine.

***Head*** (Fig. 18): dorsally smooth, no eyes visible, rostrum short, slightly downcurved.

***Antenna 1*** (Fig. 18): peduncle article 2 narrower and longer than article 1, > 2× as long as article 3; flagellum eight-articulated; accessory flagellum uni-articulated.

***Antenna 2*** (Fig. 18): peduncle articles 4 and 5 equal in length; flagellum broken off.

**Figure 19.**
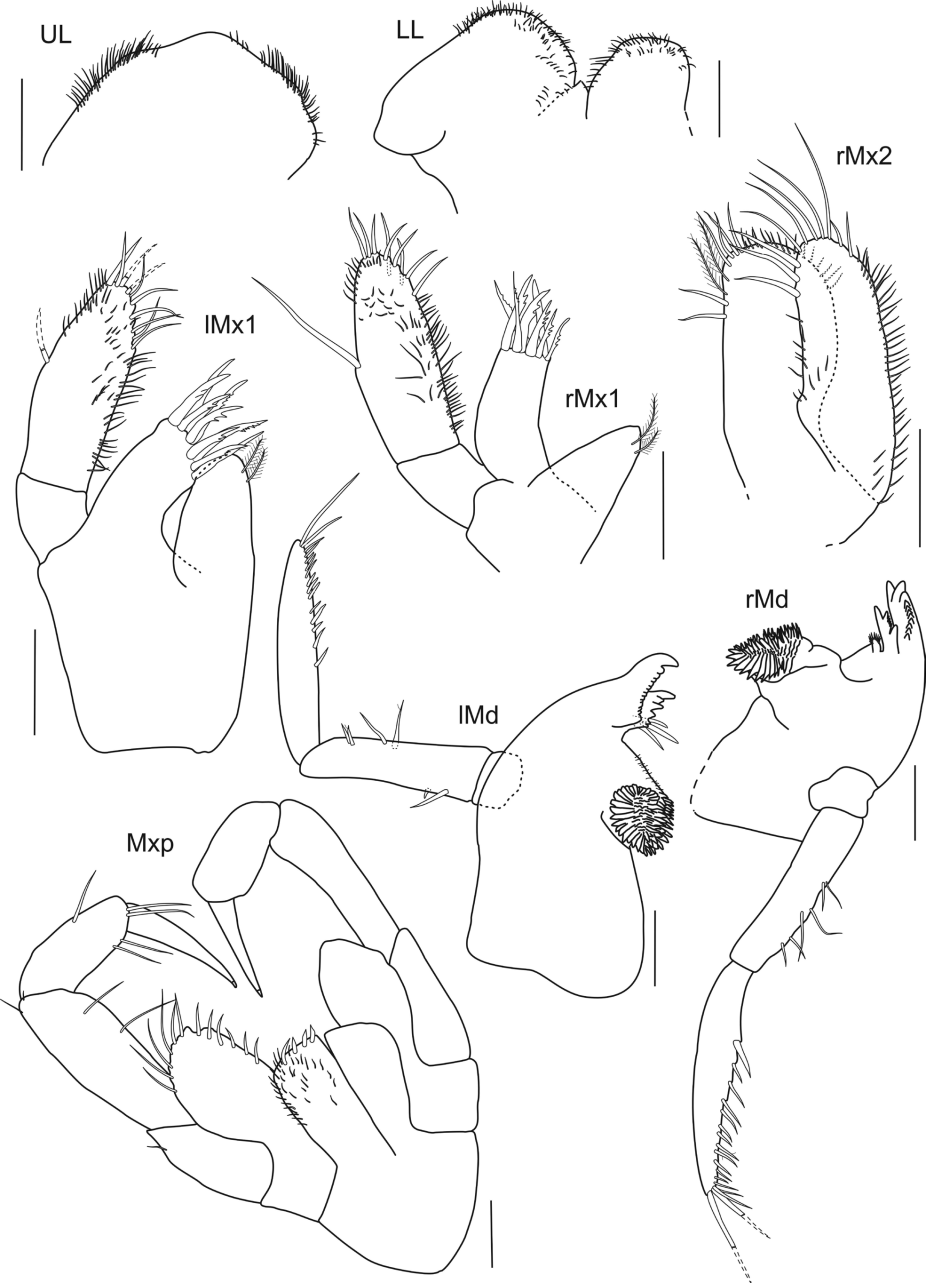
*
Rhachotropis clarionclippertoni* sp. nov., holotype, female, 8 mm, SMF 62817 (UL, LL, lMx1, rMx1, rMx2, lMnd, rMnd); Paratype, female, 8.5 mm, SMF 62818 (Mxp). Scale bars: 0.1 mm.

***Upper lip*** (Fig. 19): circular in shape, ventral margins rounded, lateral margins finely setose.

***Mandible*** (Fig. 19): incisor process strongly dentate, well-developed; left lacinia mobilis denticulate; molar process conical, edge with row of strong spines; article 3 longer than article 2, articles 2 and 3 inner margin with long slender setae, plumose setae at apical tip of palp.

***Lower lip*** (Fig. 19): setose anterodistally, outer lobes separated by broad gap.

***Maxilla 1*** (Fig. 19): inner plate bearing two subterminal plumose setae; outer plate with nine denticulate spines; article 1 of palp 1/3 length of second article, article 2 of palp with several slender setae.

***Maxilla 2*** (Fig. 19): inner and outer plates subequal in length, margins bearing stout and slender setae; inner plate slightly wider than outer plate.

***Maxilliped*** (Fig. 19): inner plate distally with three strong spines; outer plate 2× as long as inner plate, reaching almost 1/2 of article 2 of maxillipedal palp, outer plate margins and palp article setose.

**Pereon: *Gnathopod 1*** (Fig. 18): coxa 1 anteriorly extended; gnathopods similar in shape, subchelate; gnathopod 1 slightly smaller than gnathopod 2; basis bearing small spines at posteroventral corner; ischium and merus with long setae at posteroventral corner; carpal lobe extending width of propodus, spines at distal end of lobe and along posterior margin; propodus widened, oval; dactylus slender, reaching end of palm.

***Gnathopod 2*** (Fig. 18): coxa 2 subquadrate; gnathopod 2 basis 1.2× as wide as basis of gnathopod 1; ischium to dactylus similar to gnathopod 1, except carpal lobe longer and narrower.

**Figure 20.**
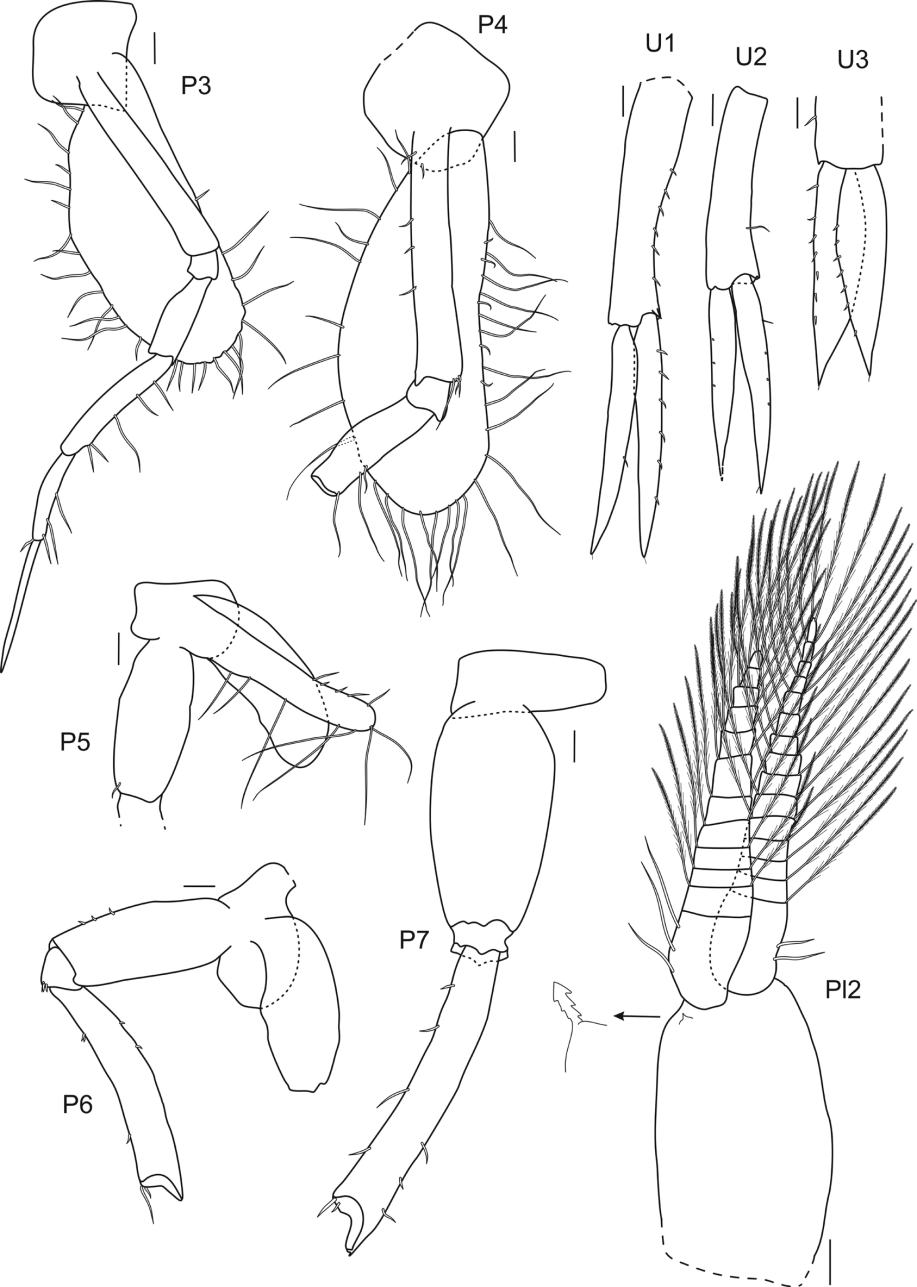
*
Rhachotropis clarionclippertoni* sp. nov., holotype, female, 8 mm, SMF 62817. Scale bars: 0.1 mm.

***Pereopods 3 and 4*** (Fig. 20): coxae 3 and 4 subquadrate. Pereopod 3 articles long and narrow; carpus, propodus and dactylus in ratio of 1:0.6:0.96. Pereopod 4 similar to pereopod 3, carpus, propodus and dactylus broken off.

***Pereopod 5*** (Fig. 20): coxa 5 lobate; basis subrectangular; remaining pereopod missing.

***Pereopod 6*** (Fig. 20): coxa 6 lobate; basis subrectangular with small posteroventral lobe; merus longer than basis; remaining pereopod missing.

***Pereopod 7*** (Fig. 20): pereopod 7 longer than pereopods 5 and 6; basis slightly expanded, subovate; merus longer than basis; rest of pereopod missing.

**Pleon: *Epimera 1–3*** (Figs 17, 18): epimeral plates ventrally rounded; epimeral plate 3 posterior margin and posteroventral corner minutely serrated.

***Pleopods*** (Fig. 20): pleopod 2 (right side) rami with 12 and 15 articles; rami similar length, as long as peduncle.

**Urosome: *Urosomites 1–3*** (Figs 17, 18): urosome segment 1 dorsolaterally with a small spine, urosomites 2 and 3 smooth.

***Uropod 1*** (Fig. 20): lanceolate; outer and inner ramus same length; rami shorter than peduncle.

***Uropod 2*** (Fig. 20): lanceolate; outer ramus shorter than inner ramus; peduncle shorter than outer ramus and longer than inner ramus.

***Uropod 3*** (Fig. 20): lanceolate; outer and inner ramus same length, nearly 2× as long as peduncle.

***Telson*** (Fig. 18): Telson > 3× as long as wide, with V-shaped cleft of ~ 15%.

##### Remarks.

The combination of the following characters found in *Rhachotropis clarionclippertoni* sp. nov. (produced first coxa, cleft telson, rostrum shorter than 1/2 of first peduncle of antenna 1) is shared with the following species: *R. americana* Bousfield & Hendrycks, 1995, *R. antarctica* K.H. Barnard, 1932, *R. gubilata* J.L. Barnard, 1964, *R. palporum* Stebbing, 1908, *R. portoricana* J.L. Barnard, 1964, and *R. rossi* Lörz, 2010, but only the new species and *R. portoricana* have no lateral teeth on pleonites 1–3. *Rhachotropis clarionclippertoni* sp. nov. is very similar to *R. portoricana* but can be distinguished by the following characters (characters of *R. portoricana* in parentheses): rounded ventrolateral margin of urosomite 1 (vs ventrolateral margin shows a drawn out process), rostrum reaching only 1/3 of the length of the cephalic lobe (vs rostrum reaching 1/2 length of the cephalic lobe), length to width ratio of basis of pereopod 6 is 0.4 (vs 0.6), length to width ratio of basis of pereopod 7 is 0.54 (vs 0.65), no posterodistal lobe on basis of pereopod 7 (vs small but distinct lobe), telson cleft 15% (vs telson cleft 8%).

##### Etymology.

The name *clarionclippertoni* refers to the Clarion-Clipperton Zone in the abyssal east Pacific, a marine area known for the high density of manganese nodules, amongst which this new amphipod was collected.

##### Distribution.

Abyssal Pacific Ocean, Clarion-Clipperton Zone in 4097–4430 m depth.

##### Molecular identification.

Following the definition given by Pleijel et al. (2008), the sequence of the holotype male of *Rhachotropis clarionclippertoni* (SMF 62817, GenBank accession number PQ734529) is designed as a hologenophore of all obtained sequences. The sequences of the paratype and additional individuals of the species are also available in GenBank (Table 2). Furthermore, the species has received a Barcode Index Number from Barcode of Life Data Systems: BOLD:AEB0147 (https://doi.org/10.5883/BOLD:AEB0147).

#### Rhachotropis laure sp. nov.

Taxon classificationAnimaliaAmphipodaEusiridae

61535576-FB6B-5413-BC62-39DA11E7E33A

https://zoobank.org/ECA944F4-D259-43D3-95E8-3A4D583BE01D

Figs 21, 22, 23, 24

##### Type material.

***Holotype***: Pacific • female, 10.5 mm, SMF 62819, COI: PQ734767, body remnants and two slides with appendages, Clarion-Clipperton Zone, BGR exploration contract area, R/V *Kilo Moana*, MANGAN 2016, EBS, Ma 16-28, 01/05/2016, 11°49.654'N, 117°00.299'W–11°49.902'N, 116°59.174'W, 4143–4133 m. ***Paratype***: Pacific • female, 10 mm, SMF 62820, COI: PQ734754, Clarion-Clipperton Zone, BGR exploration contract area, R/V *Sonne*, MANGAN 2018, EBS, SO 262-155, 09/05/2018, 11°47.436'N, 117°32.213'W–11°47.677'N, 117°47.677'W, 4352–4351 m.

##### Additional material.

Pacific • Clarion-Clipperton Zone; BGR and OMS exploration contract areas, several stations (Table 2).

##### Type locality.

Abyssal Pacific Ocean, Clarion-Clipperton Zone, 11°49.654'N, 117°00.299'W–11°49.902'N, 116°59.174'W, 4143–4133 m.

##### Description.

Based on holotype, female, 10.5 mm, SMF 62819.

**Figure 21.**
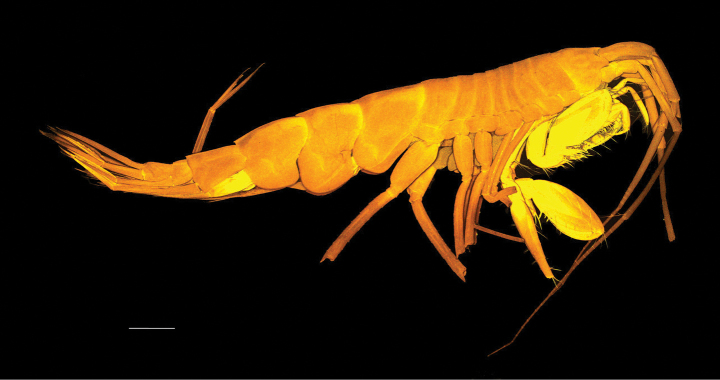
*
Rhachotropis laure* sp. nov., paratype, female, 10 mm, SMF 62820. Scale bar: 0.5 mm.

**Figure 22.**
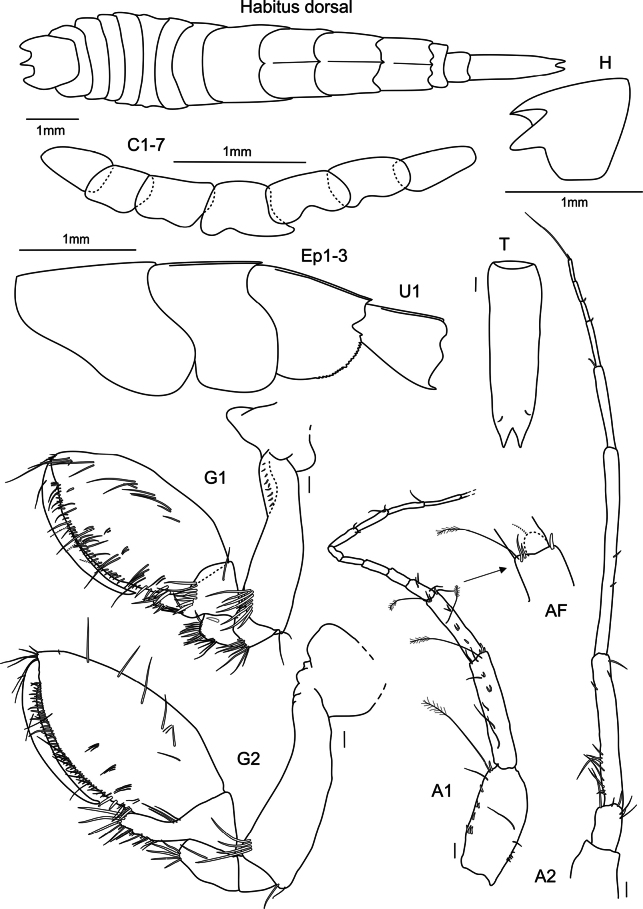
*
Rhachotropis laure* sp. nov., holotype, female, 10.5 mm, SMF 62819. Scale bars: 1 mm (Habitus, C1-7, Ep1-3, U1, H); 0.1 mm (all remaining appendages).

***Body*** (Figs 21, 22): all pereonites smooth; pleonites 1–3 with a dorsal spine, pleonites 2 and 3 with a carina.

***Head*** (Figs 21, 22): dorsally smooth, no eyes visible; rostrum short, slightly downcurved; cephalic lobes rounded.

***Antennae 1 and 2*** (Fig. 22): antennae very long, almost as long as body. Antenna 1 peduncle article 2 narrower and same length as article 1, longer than article 3; flagellum broken off; accessory flagellum uni-articulated. Antenna 2 peduncle articles 4 and 5 equal in length, several plumose setae on fourth article; article 5 long, 1/4 length of article 4.

**Figure 23.**
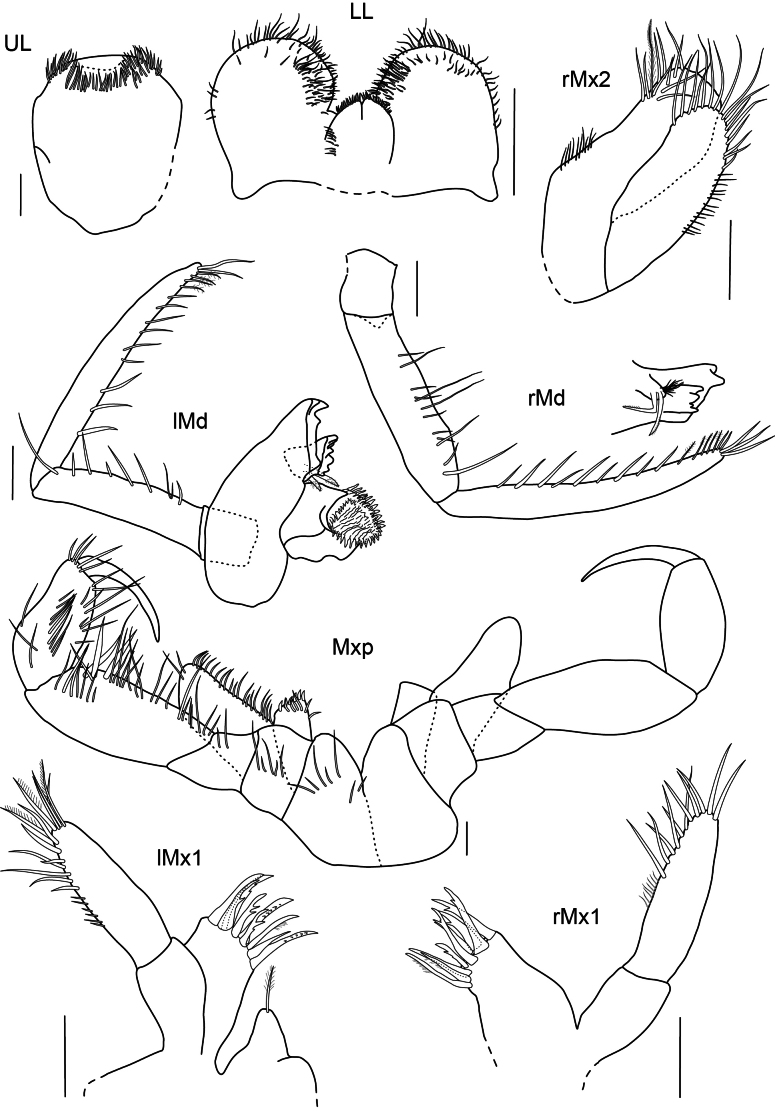
*
Rhachotropis laure* sp. nov., holotype, female, 10.5 mm, SMF 62819. Scale bars: 0.1 mm.

***Upper lip*** (Fig. 23): circular in shape, two setose areas anteriorly (ventral margin rounded, lateral margins finely setose).

***Mandible*** (Fig. 23): Mandible with incisor process well-developed; left lacinia mobilis denticulate; molar process conical, large molar, edge with row of strong spines; palp article 1 short, ~ 1/3 length of article 2, article 3 almost 2× as long as article 2, articles 2 and 3 with long slender setae, plumose setae at apical tip of left mandible palp.

***Lower lip*** (Fig. 23): setose anterodistally, outer lobes separated by broad gap.

***Maxilla 1*** (Fig. 23): inner plate bearing one subterminal plumose setae; outer plate with nine denticulate spines; article 1 of palp 1/2 length of second article, article 2 of palp with several slender setae.

***Maxilla 2*** (Fig. 23): inner and outer plates subequal in length, margins bearing stout and slender setae; inner plate slightly wider than outer plate.

***Maxilliped*** (Fig. 23): inner plate distally with short, thick spines; outer plate 2× as long as inner plate, reaching 1/2 of article 2 of maxillipedal palp, outer plate margins strongly setose; palp article strongly setose.

**Pereon: *Gnathopod 1*** (Fig. 22): coxa 1 anteriorly slightly drawn out; gnathopods similar in shape, subchelate; gnathopod 1 slightly smaller than gnathopod 2; basis bearing small spines at anteroventral corner; ischium and merus with long setae at posteroventral corner; carpus lobe extending width of propodus, spines at distal end of lobe and along the posterior margin; propodus widened, oval, palmar corner with six spines; dactylus slender, reaching end of palm.

***Gnathopod 2*** (Fig. 22): coxa 2 subquadrate; gnathopod 2 basis wider than basis of gnathopod 1; ischium to dactylus similar to gnathopod 1.

**Figure 24.**
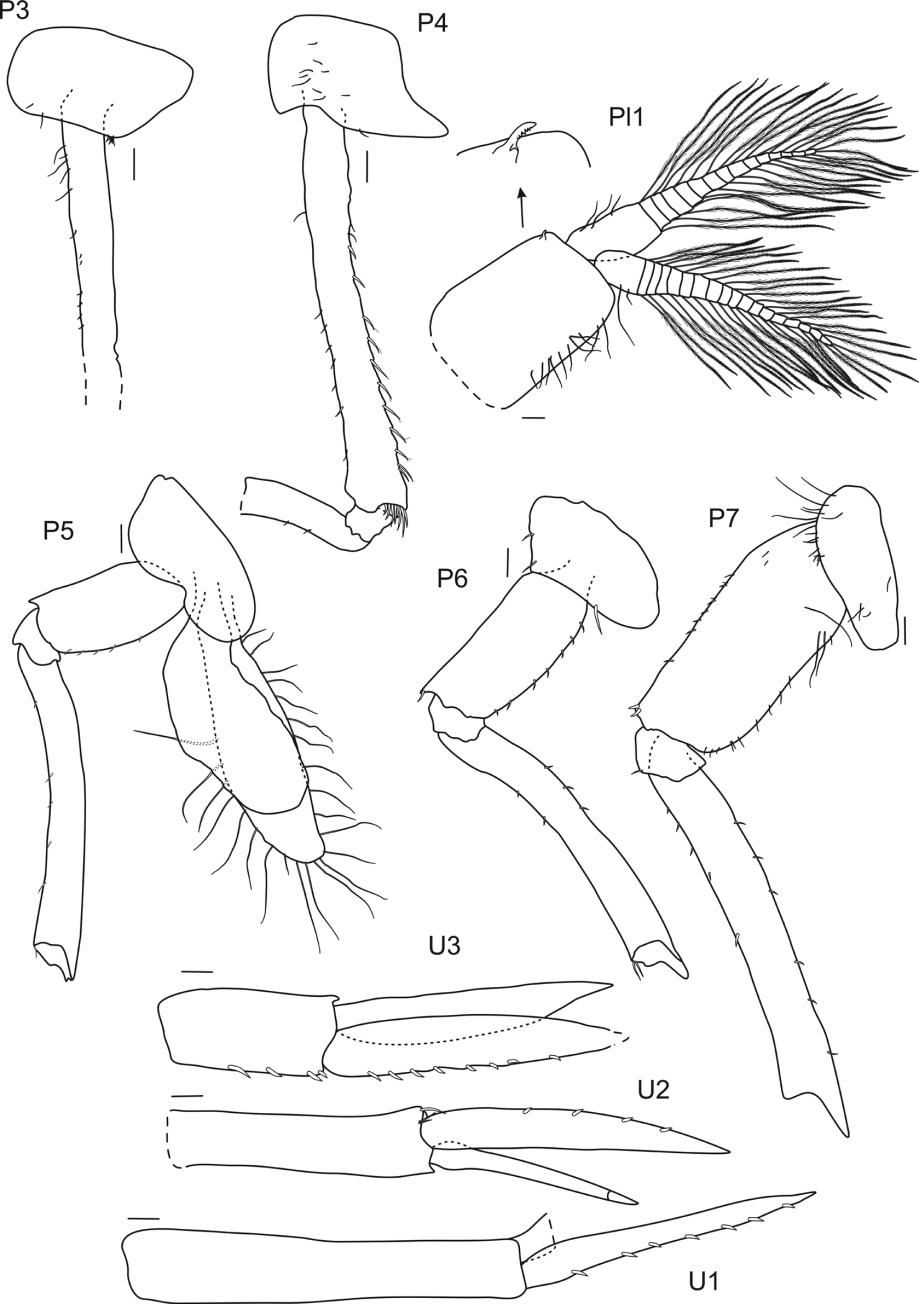
*
Rhachotropis laure* sp. nov., holotype, female, 10.5 mm, SMF 62819. Scale bars: 0.1 mm.

***Pereopods 3 and 4*** (Fig. 24): coxa 3 subquadrate, coxa 4 posteriorly drawn out. Pereopods 3 and 4 articles long and narrow; pereopod 3 broken off at basis; pereopod 4 broken off at merus.

***Pereopod 5*** (Fig. 24): coxa 5 lobate. Pereopod 5 slender, basis subrectangular, posteroventral angle notched, merus long; carpus, propodus, and dactylus broken off.

***Pereopod 6*** (Fig. 24): coxa 6 slightly lobate, pereopod 6 larger, but similar in shape to pereopod 5, merus long; carpus, propodus, and dactylus broken off.

***Pereopod 7*** (Fig. 24): coxa 7 posterior margin drawn out. Pereopod 7 longer than pereopods 5 and 6; basis with rounded at posterodistal corner; merus posteroventral angle produced, remaining pereopod missing.

**Pleon: *Epimera*** (Figs 21, 22): epimeral plates ventrally rounded, epimeral plates 1 and 2 posteriorly smooth, epimeral plate 3 serrated.

***Pleopods*** (Fig. 24): pleopod 1 (right side) rami with 15 and 18 articles; rami same length, as long as peduncle.

**Urosome: *Urosomites*** (Figs 21, 22): urosome segment 1 with carina and dorsal spine, urosomes 2 and 3 smooth.

***Uropod 1*** (Fig. 24): lanceolate; outer rami broken off; inner ramus shorter than peduncle.

***Uropod 2*** (Fig. 24): lanceolate; outer ramus 0.3× shorter than inner ramus; peduncle longer than outer ramus.

***Uropod 3*** (Fig. 24): lanceolate; inner and outer ramus approx. the same length, rami nearly 2× as long as peduncle.

***Telson*** (Fig. 22): Telson 3× as long as wide, with a V-shaped cleft of ~ 10%.

##### Remarks.

*Rhachotropis laure* sp. nov. is most similar to *Rhachotropis gislii* Thurston, 1980, *R. distincta* (Holmes, 1908), and *R. gracilis* Bonnier, 1896; these four species share the character combination of coxa 1 not being produced, cleft telson, and serrated epimeral plate 3. *Rhachotropis laure* sp. nov. can be distinguished from *R. gislii* by the latter bearing lateral teeth on the pleon, a rounded cephalic lobe, and a smooth urosomite 1. *Rhachotropis gracilis* and *R. distincta* do not show a strong excavation of coxa 4 as presented in *R. laure* sp. nov. Characters separating the morphologically close *Rhachotropis* species are listed in Table 6.

**Table 6.** Morphological comparison of *Rhachotropis laure* sp. nov. with similar known species.

**Character/Species**	*** Rhachotropis gislii* Thurston, 1980**	*** Rhachotropis distincta* (Holmes, 1908)**	*** Rhachotropis gracilis* Bonnier, 1896**	*** Rhachotropis arii* Thurston 1980**	*** Rhachotropis laure* sp. nov**.
C4 excavate behind	slightly	slightly	no	yes, noticeably	yes, noticeably
Pleonites, lateral teeth	yes, on Pl1-Pl2	no	no	no	no
Urosomite 1 toothed	no	yes, longer	yes	yes	yes, small
Epimeral plate 3	serrated	serrated	serrated	smooth	serrated

Another morphologically very similar species to *Rhachotropis laure* sp. nov. is *Rhachotropis arii* Thurston, 1980, but the latter has a smooth third epimeral plate, whereas in *R. laure* sp. nov. it is distinctly serrated (Table 6).

##### Etymology.

This species is named after Laure de Montety who works at the Marine and Freshwater Research Insitute of Iceland, Hafrannsóknastofnun, for illuminating many benthic organisms with her taxonomic expertise and for being a friend. It is used as a noun in apposition.

##### Distribution.

Abyssal Pacific Ocean, Clarion-Clipperton Zone in 4026–4368 m depth.

##### Molecular identification.

Following the definition given by Pleijel et al. (2008), the sequence of the holotype male of *Rhachotropis laure* (SMF 62819, GenBank accession number PQ734767) is designed as a hologenophore of all obtained sequences. The sequences of the paratype and additional individuals of the species are also available in GenBank (Table 2). Furthermore, the species has received a Barcode Index Number from Barcode of Life Data Systems: BOLD:AEB2577 (https://doi.org/10.5883/BOLD:AEB2577).

## Discussion

Herewith we provided descriptions of five species in three eusirid genera. Within the family Eusiridae, the genus *Cleonardo* Stebbing, 1888 contains ten species, with four of these found in the Pacific. The present addition of two more species, of which one, *C. compassionate* sp. nov., is distributed both in central East Pacific and NW Pacific (one of the haplotypes of the COI is shared between the two regions), indicates high mobility in these amphipods.

The genus *Dorotea* Corbari, Frutos & Sorbe, 2019 contains two species (Horton et al. 2024), one of which was recorded from tropical South West Pacific, while the other was from the sub-Antarctic part of the Indian Ocean. The present finding is only the third record in the genus. Previously *Dorotea* was known only from the shelf (*D. aberrantis*, Indian Ocean) and upper bathyal (*D. papuana*, tropical West Pacific) (Bellan-Santini and Ledoyer 1987; Corbari et al. 2019). The addition of this third species extends the geographic distribution of the genus and also increases the bathymetric range to the abyss.

*Rhachotropis* Smith, 1883 is by far the largest genus of the family Eusiridae, with 67 described species, of which 30 have been collected from the Pacific (Corbari et al. 2024; Horton et al. 2024). *Rhachotropis* species are found in all oceans and major basins of the world: Arctic, Atlantic, Mediterranean, Caribbean, Indian, Pacific, and the Southern Ocean. They have been collected in all water depths, from the shelf (e.g., Weisshappel 2000; d’Udekem d’Acoz et al. 2007; Lowry and Springthorpe 2005) to the abyss (Thurston 1980; Lörz 2010), and in trenches (Dahl 1959; Lörz et al. 2018). As a result, this genus has the widest horizontal and vertical distribution among all amphipod genera (Lörz 2010; Lörz et al. 2012, 2018, 2023).

## Supplementary Material

XML Treatment for Cleonardo

XML Treatment for Cleonardo daniela

XML Treatment for Cleonardo compassionate

XML Treatment for Dorotea

XML Treatment for Dorotea elizae

XML Treatment for Rhachotropis

XML Treatment for Rhachotropis clarionclippertoni

XML Treatment for Rhachotropis laure

## Additional information

### Author ORCIDs

Anne-Nina Lörz https://orcid.org/0000-0001-9942-349X

Laura Engel https://orcid.org/0009-0009-9841-9590

Halina Jereczek https://orcid.org/0009-0004-6132-1841

Nikodem Ćwierz https://orcid.org/0009-0003-2311-2021

Anna M. Jażdżewska https://orcid.org/0000-0003-2529-0641

### Data availability

All of the data that support the findings of this study are available in the main text.
